# New insights into the genome of *Rhodococcus ruber* strain Chol-4

**DOI:** 10.1186/s12864-019-5677-2

**Published:** 2019-05-02

**Authors:** Govinda Guevara, Maria Castillo Lopez, Sergio Alonso, Julián Perera, Juana María Navarro-Llorens

**Affiliations:** 1 Departamento de Bioquímica y Biología Molecular, Facultad de Ciencias Biológicas, Universidad Complutense de Madrid, Madrid, Spain; 2 Program of Predictive and Personalized Medicine of Cancer (PMPPC), Germans Trias i Pujol Research Institute (IGTP), Carretera de Can Ruti S/N 08916 Badalona, Barcelona, Spain

**Keywords:** *Rhodococcus ruber*, Catabolism, Biodegradation, Genome analysis

## Abstract

**Background:**

*Rhodococcus ruber* strain Chol-4, a strain isolated from a sewage sludge sample, is able to grow in minimal medium supplemented with several compounds, showing a broad catabolic capacity. We have previously determined its genome sequence but a more comprehensive study of their metabolic capacities was necessary to fully unravel its potential for biotechnological applications.

**Results:**

In this work, the genome of *R. ruber* strain Chol-4 has been re-sequenced, revised, annotated and compared to other bacterial genomes in order to investigate the metabolic capabilities of this microorganism. The analysis of the data suggests that *R. ruber* Chol-4 contains several putative metabolic clusters of biotechnological interest, particularly those involved on steroid and aromatic compounds catabolism.

To demonstrate some of its putative metabolic abilities, *R. ruber* has been cultured in minimal media containing compounds belonging to several of the predicted metabolic pathways. Moreover, mutants were built to test the naphtalen and protocatechuate predicted catabolic gene clusters.

**Conclusions:**

The genomic analysis and experimental data presented in this work confirm the metabolic potential of *R. ruber* strain Chol-4. This strain is an interesting model bacterium due to its biodegradation capabilities. The results obtained in this work will facilitate the application of this strain as a biotechnological tool.

**Electronic supplementary material:**

The online version of this article (10.1186/s12864-019-5677-2) contains supplementary material, which is available to authorized users.

## Background

*Rhodococci* belong to the taxon of nocardioform actinomycetes. These aerobic Gram-positive bacteria are found in diverse environmental niches and are world-widely distributed being abundant in soil, water and marine environments [1]. They differ from other Actinomycetes and are called the Mycolata because their distinctive cell envelope contains large branched chain lipids known as mycolic acids [2]. The genome size of these non-sporulating mycolic-acid-containing bacteria varied for different strains from 4.3 Mb (e.g. *R. rhodnii* strain LMG5362, [3]) to 10.0 Mb (e.g. *R. wratislaviensis*, GCA_000583735.1).

*Rhodococci* are known for displaying a wide metabolic versatility and for their ability to transform a varied range of pollutants such as aliphatic and aromatic hydrocarbons, oxygenated and halogenated compounds, nitroaromatics, heterocyclic compounds, nitriles, and various pesticides [4]. The analysis of their genomes has revealed a multiplicity of genes, a high genetic redundancy of metabolic pathways, and a complex regulatory network [5]. Moreover, some *Rhodococcus* strains harbor circular and linear plasmids that contain genes encoding additional catabolic enzymes [6–8]. Even the extracellular polysaccharides of the outer membrane of rhodococci contribute to the catabolism of aromatic compounds [9]. This versatile metabolic capacity and also their environmental persistence and tolerance to stress conditions make *Rhodococcus* strains good candidates for biotechnological processes such as bioremediation, biotransformations or biocatalysis [4, 10, 11]. On the other hand, *Rhodococcus* strains are able to synthesize compounds of industrial interest including biosurfactants [12] and steroid precursors [13]. For all these reasons, the characterization of different *Rhodococcus* metabolic capabilities is necessary to fully exploit their biotechnological potential.

*Rhodococcus ruber* strain Chol-4, isolated from a sewage sludge sample, is classified as a Gram-positive bacteria belonging to the actinobacteria taxon with a high guanine-cytosine content [14]. This strain is able to grow in minimal medium supplemented with several aromatic compounds, showing a broad catabolic capacity. We have recently published the draft genome sequence of this bacterium [15]. The analysis of the genomic sequence of different bacterial species, i.e. the presence of specific genes or gene families, allows inferring their particular metabolic capabilities. In this work we present novel and more comprehensive results, both computational and experimental, that support the versatile metabolic potential of *R. ruber* strain Chol-4.

## Methods

### Bacterial strains and culture conditions

The bacterial strains and plasmids used in this work are listed in Additional file 1.

*E. coli DH5α* was purchased to Thermo Fisher Scientific*. E. coli GM48* was obtained from the *E. coli* Genetic Resources Collection (CGSC5127 number) and *E. coli S17.1* was obtained from the ATCC Bacteriology Collection (ATCC 47055 number). *Rhodococcus ruber strain Chol-4,* a strain isolated from a sewage sludge sample [14], and their derived mutants have been obtained in our laboratory.

*Escherichia coli* cells were grown at 37 °C in Luria Bertani (LB) [16]. *Rhodococcus ruber* and its derived mutant strains were routinely grown in LB or minimal medium (Medium 457 of the DSMZ, Braunschweig, Germany) containing the desired carbon and energy source under aerobic conditions at 30 °C in a rotary shaker (250 rpm) for 1–3 days. Where appropriate, antibiotic were added at the following concentrations: ampicillin (100 μg/mL), nalidixic acid (15 μg/mL) or kanamycin (25–50 μg/mL for *E. coli* or 200 μg/mL for *Rhodococcus*). For the growth experiments, a LB pre-grown culture was washed two times with minimal medium prior to inoculation of 10 mL of fresh minimal medium (initial DO_600nm_ = 0.05) supplemented with an organic compound as only source of energy and carbon. Volatile compounds such as indane, tetralin, isopropanol, 1,3-butanediol, 2,3-butanediol, xylene, benzene, ethylbenzene, toluene, phenylacetic acid or styrene were provided supplied in gas phase via saturated atmosphere (Additional file 2). Aromatic compounds were used at 1 mg/mL of naphthalene in powder, 10 mM sodium benzoate, 2 mM phenol, 5 mM L-tryptophan, 4 mM vanillic acid, 4 mM gentisate, 5 mM homogentisate, 2 mM catechol, 2.2 mM cholic acid, 2 mM DHEA and 10 mM protocathecuate, 15 mM biphenyl, 20 mM phthalate, 5 mM 2-aminobenzoate, from 2 to 4 mM salicylic acid, 0.5 mM hydroxyquinol or 2 mM L-tyrosine. DHEA (dehydroepiandrosterone) and cholic acid were previously dissolved in 16.5 mM methyl-β-cyclodextrin to form inclusion complexes following a modification of a previously reported method [17] and prepared as described [18]*.* Although it is not necessary to add methyl-β-cyclodextrin to dissolve cholic acid at the concentrations employed in our experiments, we have used them in the cholic acid growth experiments to homogenize the experimental conditions for compounds with similar structures (e.g. steroids). Biological replicas (2 to 5 replicates) were performed for all growth experiments.

Competent and electrocompetent cells of *E. coli* were prepared and transformed as previously described [16]. Selection of transformed cells was carried out in LB agar plates supplemented with appropriate antibiotics.

### DNA manipulation and sequencing

Chromosomal DNA extraction from *R. ruber* strain Chol-4 was performed using the Cetyl Trimethyl Ammonium Bromide procedure [19]. Briefly, bacterial cells were collected from a LB plate, resuspended in 400 μL Tris-EDTA buffer (10 mM Tris/HCl, pH 8, 1 mM EDTA) and incubated at 80 °C for 20 min. Then, 50 μL of lysozyme (100 mg/mL) was added and incubated at 37 °C for 12 h. Afterwards, 70 μL of 10% SDS and 5 μL of proteinase K (10 mg/mL) were added and the sample was incubated for 10 min at 65 °C. Proteins were precipitated with 100 μl of 5 M NaOH and 100 μl CTAB (0.1 g/ml resuspended in 0.7 M NaOH) for 10 min at 65 °C. DNA was purified by extraction with chloroform-isoamyl alcohol (24:1) and phenol-chloroform-isoamyl alcohol (25:24:1) and precipitated with 0.6 vol of isopropanol at room temperature for 30 min. After centrifugation, DNA was washed with 70% ethanol and resuspended in sterile water.

Manipulation of genomic DNA was carried out according to standard protocols [16], and the extracted DNA was purified three times to achieve highest purity and quality for subsequent sequencing of the complete genome.

Two independent NGS experiments were combined to generate this new version of the *R. ruber* Chol-4 de novo genomic assembly. One was previously performed using Roche 454 technology [15]. A new one based on massively parallel pyrosequencing of the genomic DNA was done by Biejing Genomics Institute, BGI - Hong Kong Laboratory (Hong Kong, China), using Illumina HiSeq 2000 platform. A 500 bp short-insert library was constructed and a 91 PE sequencing was used as strategy. Before data delivery, Incoming Quality Control and three levels of Quality Control processes (e.g. GC content and depth correlative analysis) were performed by BGI.

The program SPAdes v3.1.0 [20] was employed to assemble the reads. This assembler accepts different formats for the input sequences (Fasta, FastaQ, single-end, pair-end, etc.), thus allowing the combination of sequences generated by different sequencing platforms. Four large sequences previously generated in our lab by conventional cloning and Sanger sequencing (JQ083440.1, JQ083439.1, EU878550.1 and FJ842098.2) were entered as trusted contigs (−-trusted-contigs flag), Illumina reads as paired-reads and Roche reads as unpaired. To reduce the number of mismatches and short indels, mismatch corrector was run after the initial assembly by specifying the flag --careful in the SPAdes command. The quality assessment of the genome assembly was done using QUAST [21]. Manual curation of the assembly was subsequently carried out in order to reduce the number of contigs, based on their length, the G + C content and sequence similarity of the generated contigs with other known species.

### Mutagenesis of *R. ruber* strain Chol-4

Unmarked gene deletions were carried out as described previously in *R. erythropolis* SQ1 involving conjugative transfer of a mutagenic plasmid carrying the *sacB* selection system [22]. Specific sets of primers were designed from the up and downstream sequences of each cluster (ketoadipate and naphtalen pathway). Polymerase chain reaction (PCR) amplicons were obtained from isolated *R. ruber* strain Chol-4 genomic DNA. Primers and conditions employed in the experiments are summarized in Additional file 3. To facilitate cloning, the primer sequences included restriction sites: the ketoadipate cluster contained *Eco*RI-*Xba*I for the up fragment, and *Xba*I-*Hin*dIII for the down fragment; the naphtalen cluster contained *Xho*I*-Hind*III and *Xba*I-*Hin*dIII for the up and down fragment, respectively.

PCR amplicons (up and down fragments) were first cloned separately into pGEM-T-Easy vectors and then combined in order to get an *Eco*RI-*Hin*dIII and *Xho*I-*Hind*III fragments containing a truncated cluster. Transformation into *E.coli* GM48 was necessary in order to avoid dam methylation of the *Xba*I site. The *Eco*RI-*Hind*III and *Xho*I-*Hind*III inserts, containing the fused up and down fragments, were transferred to pK18mobsacB plasmid [23] to construct the mutagenic plasmid pK18(U + D) used for the partial deletion of the corresponding cluster from *R. ruber* strain Chol-4 chromosome.

Every mutagenic plasmid was introduced into *E. coli* S17.1 and mobilized to *R. ruber* strain Chol-4 by conjugation as previously described [19]. *R. ruber* transconjugants that had integrated the plasmid by homologous recombination were selected on LB plates supplemented with nalidixic acid. The cluster fragment deletion was achieved as a result of a second spontaneous homologous recombination process within the genome of *R. ruber* strain Chol-4. Colony PCR detection was performed to confirm the deletion in the *nar* and *pca* clusters in the mutant *R. ruber* strains.

### Genome analysis and annotation

Homology searches were performed using the BLAST server of the NCBI (http://blast.ncbi.nlm.nih.gov/Blast.cgi). The annotation of the genome was carried out using the GenBank tool PGAP and the on-line service RAST (http://rast.nmpdr.org/). The complete genome sequence has been deposited at GenBank under accession number NZ_ANGC00000000.2. The program Circos was used to visualize genomic data [24].

### Pulsed field gel electrophoresis (PFGE)

PFGE was performed from 10 mL of a cell culture grown at OD_600nm_ of 0.8–1.0. Cells were collected by centrifugation and suspended in 0.5 mL of cell suspension solution (10 mM Tris-HCl pH 7.2, 20 mM NaCl, 100 mM EDTA). Plugs containing the cells were prepared with 1.5% agarose, placed in lysis buffer (1 mg/mL lysozyme, 10 mM Tris-HCl pH 7.2, 50 mM NaCl, 100 mM EDTA, 0.2% DOC, 0.5% N-laurylsarcosine sodium salt, 0.06 g/L RNase) and incubated for 1 h at 37 °C with soft shaking. Lysis was followed by two washes in 20 mM Tris-HCl pH 8 and 50 mM EDTA. The plugs were placed in 3 mL proteinase solution (1 mg/mL proteinase K, 100 mM EDTA pH 8.0, 1% N-lauryilsarcosine sodium salt, 0.2% DOC) and incubated with gently shaken at 42 °C for 18 h. After removing the proteinase solution, 9 mL TE containing 40 μg/mL PMSF were added and kept at 50 °C for one hour, repeating the whole process two times. After washing twice for 15 min in 20 mM Tris-HCl pH 8, 50 mM EDTA the DNA in plugs was resolved by PFGE on a contour-clamped homogeneous electric field II Mapper system (Bio-Rad Laboratories) in 0.5× Tris-borate-EDTA and the following running conditions: 6 V/cm for 18–24 h at 13 °C, with a 50-s switch time. Gels were stained in Gel Red solution (5 min) and photographed under UV light.

### Phytosterol consumption followed by mass spectrometry- high performance liquid chromatography (MS-HPLC)

*R. ruber* was grown at 30 °C with 200 rpm shaking, in 25 mL of minimal medium (M457 of the DSMZ, Braunschweig, Germany) supplemented with a mixture of industrial phytosterols in powder (around 0.7 mg/mL), kindly given by Gadea S.A. Two mL aliquots were collected at different times and 1 mg of pregnenolone was added as internal control of the extraction. The steroid fraction was extracted twice with 2 mL of chloroform. HPLC and MS determination was carried out in the Chromatography Service of the Biological Research Center (“Centro de Investigaciones Biológicas” CIB-CSIC). The relative peak area was calculated as the ratio between the HPLC peak area obtained for each phytosterol (brassicasterol, campesterol, stigmasterol and β-sitosterol) and the peak area of pregnenolone used as internal control. The experiment was done twice.

## Results

### General genome features

*R. ruber* strain Chol-4 genome was sequenced using two different Next Generation Sequencing (NGS) technologies. First, a genomic library was generated and sequenced in a Roche 454 GS FLX instrument. After quality filtering and adapter clipping, this library rendered 242,042 reads with an average length of 400 bp [15]. A second library was independently generated and processed by pair-end sequencing in an Illumina HiSeq 2000 instrument (see methods). This library generated 2,782,965 pair end reads of 90 bp with an average fragment size of 500 bp between pairs. Single-reads from the 454 library and pair-end reads from the Illumina library were combined with four larger sequences (6.3 to 11.7 Kb) that we previously obtained by standard cloning and Sanger sequencing (GenBank accession numbers JQ083440.1, JQ083439.1, EU878550.1 and FJ842098.2). All the sequences were assembled using SPAdes de novo assembler v3.1.0 [20]. The initial assembly generated 129 sequence scaffolds between 128 bp and 1,025,475 bp covering 5.63 Mb, with N50 of 438,623 bp and L50 of 4). These scaffolds were named successively according to their length, being Scaffold_001 the longest and Scaffold_129 the shortest (Additional file 4). This nomenclature was maintained in the final version of the assembly uploaded to GenBank (NZ_ANGC00000000.2). The vast majority (*n* = 126) of these scaffolds were composed of a single sequence *contig* with no internal gaps. Hence, for all practical purposes, ‘scaffold’ and ‘*contig’* denominations would be interchangeable in this work. To streamline the final assembly, twenty scaffolds shorter than 500 bp, and covering less than 3.5 kb in total, were discarded. Of the remaining 109 scaffolds, 65 (161.2 kb) exhibited a G + C content below 55%, and sequence similarity to plasmid vectors and genomes from unrelated species. These scaffolds presumably originated from cross-contamination of the NGS experiments, where several libraries were sequenced in parallel in the same flow cell, and therefore they were removed from the final assembly. The remaining 44 scaffolds of the final assembly exhibited a high G + C content (70.7%) typical of Rhodococci, and very high sequence similarity to other *Rhodococcus* genomes. These 44 scaffolds covered 5.46 Mb with N50 of 438,623 bp, L50 of 4, and an average read depth above 100X. These sequences are available from the NCBI GenBank database under the accession numbers NZ_ANGC00000NNN.1, where NNN indicates the scaffold number. We provide a more detailed report of the assembly in Additional file 5.

**Fig. 1:**
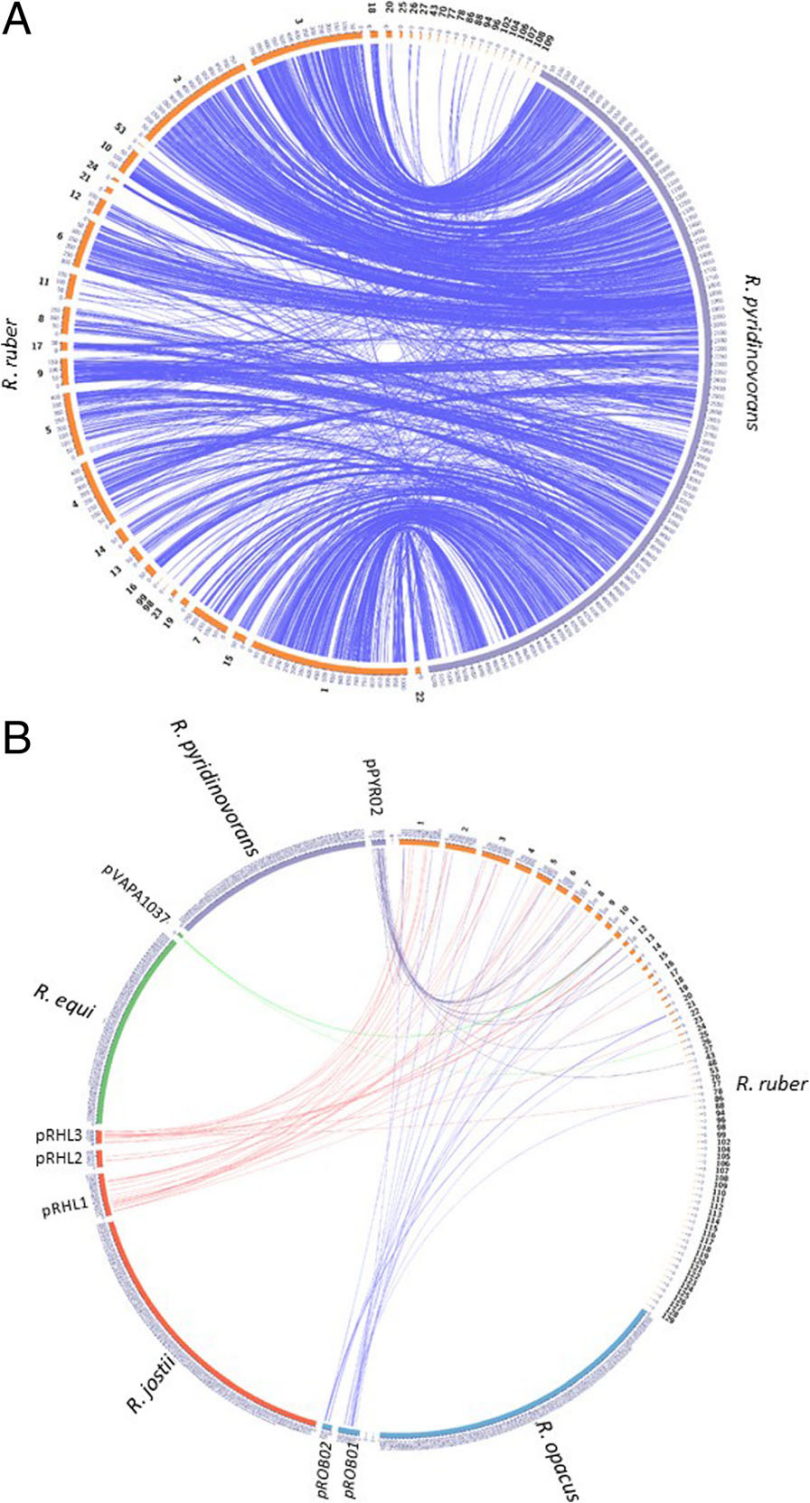
Sequence homology of the 44 scaffolds of *R. ruber* strain Chol-4 genome assembly (in orange) with the genome of closely related *Rhodococcus* bacteria. In **a** the comparison with the chromosome of its closest relative, *R. pyridinivorans* (in purple). *R. ruber* strain Chol-4 scaffolds are ordered and oriented according to their probable genomic location assuming a high level of synteny between these two microorganisms. The internal blue edges indicate regions of sequence homology above 70% with a minimum length of 0.5 kb. Scaffold order and orientation were computed to minimize de number of cross-overs among edges. In **b** comparison with the chromosomes and extrachromosomic elements of *R. pyridinivorans* (purple), *R. equi* (green)*, R.jostii* (red) *and R. opacus* (blue). This color code is also use for the internal edges indicating regions of sequence homology above 70% with a minimum length of 0.5 kb. For simplicity, only the homology with extrachromosomal elements is shown. *R. ruber* strain Chol-4 genome scaffolds are ordered according to their length. Many of the sequences found in the large plasmids pPYR02 (*R. pyridinovorans*), pVAPA1037 (*R. equi*), pRHL1–3 (*R. jostii*) and pROB01–02 (*R. opacus*), are also present in the genome of *R. ruber* strain Chol-4. In both A and B, the small font numbers outside the scaffolds indicate their internal coordinate, in kb. The large font numbers indicate the chromosome, plasmid or scaffold names as they appear in the original GenBank entries

Figure 1a shows the DNA sequence similarity between *R. ruber* Chol-4 genome and *R. pyridinivorans* SB3094 (GenBank: NC_023150.1), one of its closest relatives. The figure provides an approximation of how the *R. ruber* contigs might be arranged along its genome assuming a high degree of synteny with *R. pyridinovorans*. No evidence of circular plasmids in the genome of *R. ruber* Chol-4 was found by pulsed-field gel electrophoresis. However, some genetic elements present in other *Rhodococcus* plasmids were found interspersed along the Chol-4 genome (Fig. 1b).

### Annotation of *Rhodococcus ruber* strain Chol-4

Genome annotation using RAST server [25] identified 5049 coding sequences and 59 RNAs. 53 out of the 59 RNAS are tRNAs representing 43 different anticodons are encoded in the *R. ruber* genome (Additional file 6). There were at least 7 tRNAs in multicopy: tRNA^Met^ (ATG) is present in 4 copies, tRNA^Gly^ (GGC) in 3 copies and tRNA^Val^ (GTC), tRNA^Glu^ (GAG), tRNA^Ala^ (GCC), tRNA^Asp^ (GAC) and tRNA^Leu^ (CTC) in 2 copies. The codon usage correlated with the high G + C content of this strain as G + C codons are predominant in this organism (Additional file 6). Codons that have a T at the third position lacked a cognate tRNA in *R. ruber* with the single exception of Arg (CGT).

The number of tRNAs was similar to others *Rhodococcus* strains that display a median value of 53 tRNAs although there are exceptions such as *R. rhodnii* ASM72037v1 that contains 69 tRNAs (EMBL: NZ_JOAA00000000.1). The genomic assembly revealed a single *rrn* operon located in the NZ_ANGC02000002.1 contig containing the genes for 16S, 23S and 5S rRNA.

The 4861 protein-coding detected ORFs covered nearly 91% of the genome. Among the coding sequences, the analysis revealed at least 129 genes related to the metabolism of aromatic compounds: 15 of them involved in peripheral degradation pathways (quinate, benzoate and *p*-hydroxibenzoate degradation), 15 genes related to the aromatic amine catabolism and 6 genes associated with the gentisate degradation pathway. A small number of genes were involved in the resistance to antibiotics (resistance to vancomycin, fluoroquinolones, β-lactamase), while a relatively large number of genes were related to the resistance to toxic compounds, such as mercury and arsenic.

### Mobile elements

Within the genome of the strain Chol-4 we found a few mobile elements (Additional file 7), some of them remaining as pseudogenes. Surprisingly, 25% of these mobile elements were concentrated in a 120 kb region of NZ_ANGC020000011.1 contig.

*R. ruber* Chol-4 genome had two copies of IS*1164* from the IS*256* transposase family (the prototype of a major family of bacterial insertion sequence elements) in NZ_ANGC02000007.1 contig. This element has also been found in other *Rhodococcus* strains [5]. In the same contig there were two genes (D092_RS18300 and D092_RS17945) coding for an identical protein keeping a 90–92% base identity with elements of *Rhodococcus pyridinivorans* SB3094 plasmid (CP006997.1) and with elements of the pNSL1 plasmid (KJ605395.1) from *Rhodococcus sp.* NS1. Both genes share 84% identity with a transposase of *Mycobacterium sp.*

Other two IS elements, described for some *Rhodocococcus,* were absent in this genome: the IS*2112* element belonging to the IS*110* family found in *R. rhodochrous* NCIMB 13064 and related to genome rearrangements [26] and the IS*1166* element from the IS*256* family, found in *R. erythropolis* IGTS8 [27].

Apart from those derived of mobile elements, strain Chol-4 contains many different recombinases with different putative roles (Additional file 8).

### Other genetic elements

**Fig. 2:**
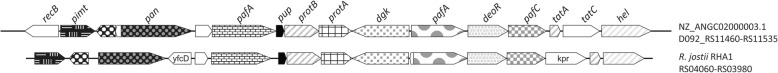
Pup proteasome in *R. ruber*. Abbreviations: *recB*: RecB family exonuclease; *pimt:* protein-L-isoaspartate methyltransferase; *pan*: bacterial proteasome-activating AAA-ATPase; *pafA*: proteasome accessory factor, Pup ligase PafA’ paralog, possible component of postulated heterodimer PafA-PafA’; *pup:* prokaryotic ubiquitin-like protein Pup; *protA* and *protB*: proteasome subunit α and β bacterial; *dgk*: diacylglycerol kinase; *deoR*: putative DeoR-family transcriptional regulator; *pafC*: DNA-binding protein; *tatA* and *tatC*: twin-arginine translocation proteins; *hel*: DEAD/DEAH box helicase; *yfcD*: nudix hydrolase YfcD; *kpr*: 2-dehydropantoate 2-reductase. All *R. jostii* RHA1 genes have the prefix “RHA1_” not included in the figure

Some actinobacteria such as *Mycobacterium tuberculosis* contain from 1 to 3 clustered regularly interspaced short palindromic repeats (CRISPR) elements (CRISPR database, https://crispr.i2bc.paris-saclay.fr/crispr/) [28]. However, *R. ruber* apparently is a strain devoid of detectable CRISPRs systems, similarly to other *Rhodococcus* strains. On the other hand, we found a gene cluster related to specialized protein degradation systems that includes a 20S proteasome activity (subunits α and β), an ATPase (that use ATP to unfold proteins and translocate them into the proteasome) and a system of tagging proteins for degradation with Pup prokaryotic ubiquitin-like protein. Conjugation with Pup serves as a signal for degradation by the mycobacterial proteasome (Fig. 2) [29, 30]. Most of the restriction modification systems detected in this genome are classified as type I or IV (Additional file 9).

### Aromatic compounds specific gene clusters

**Fig. 3:**
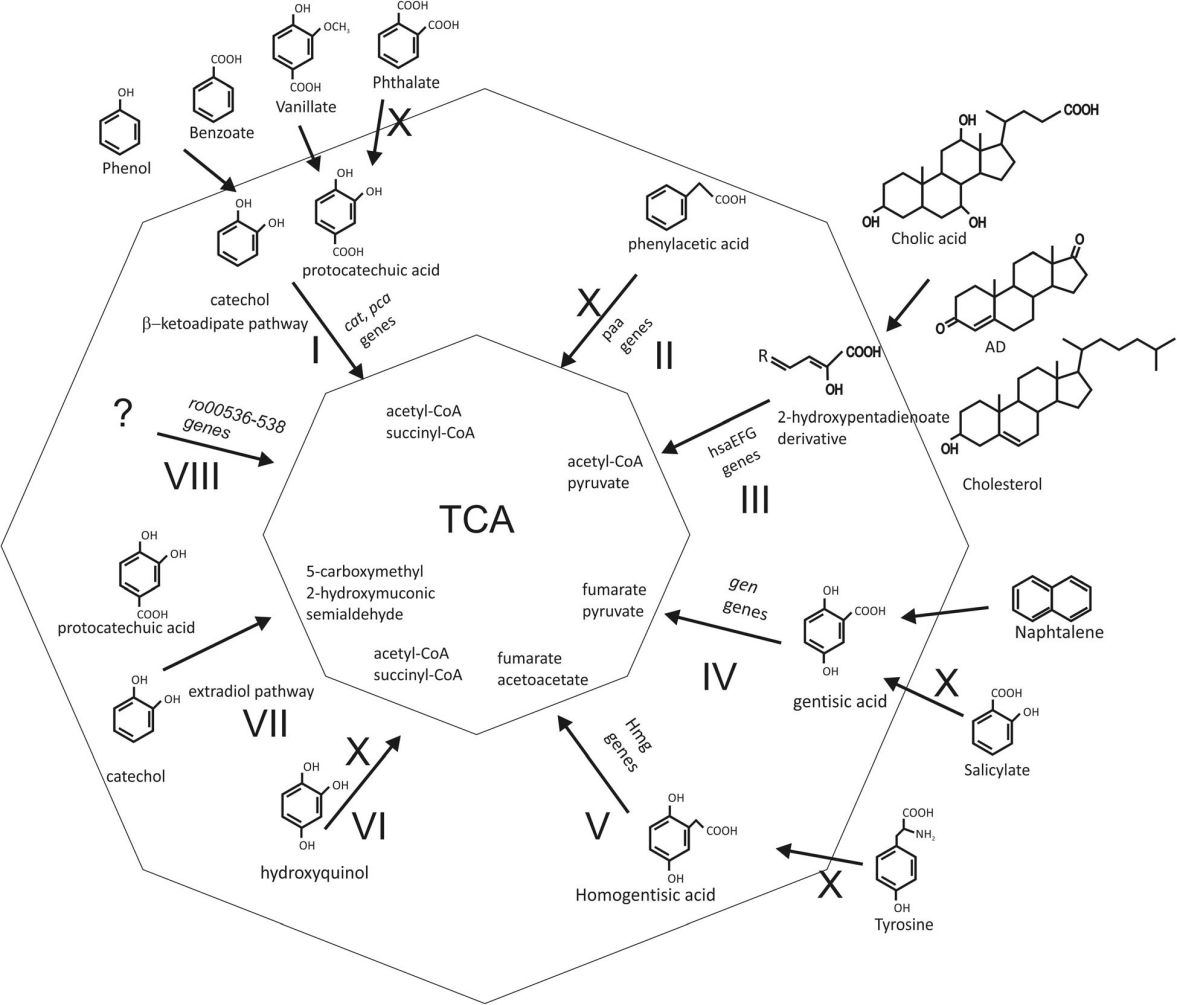
Aromatic compounds metabolism. Scheme of the aromatic compounds catabolic pathways: **I)** β-ketoadipate pathway, **II)** phenylacetate pathway, **III)** 2-hydroxypentadienoate pathway, **IV)** gentisate pathway, **V)** homogentisate pathway), **VI)** hydroxyquinol pathway, **VII)** homoprotocatechuate pathway, and **VIII)** a pathway found in *R. jostii* RHA1 comprising a hydroxylase, an extradiol dioxygenase, and a hydrolase. Peripheral pathways are depicted outside the external ring. The “X” indicates the inability of *R. ruber* to grow in the presence of these compounds as single source of carbon and energy

The *R. ruber* Chol-4 genome annotation revealed the presence of a rich set of gene cluster that may code for several aromatic compounds catabolic pathways, reflecting a high potential for degrading this kind of compounds. The catabolism of aromatic compounds proposed for *R. ruber* is outlined in Fig. 3 showing the peripheral, central and basic pathways.

### Central pathways

**Fig. 4:**
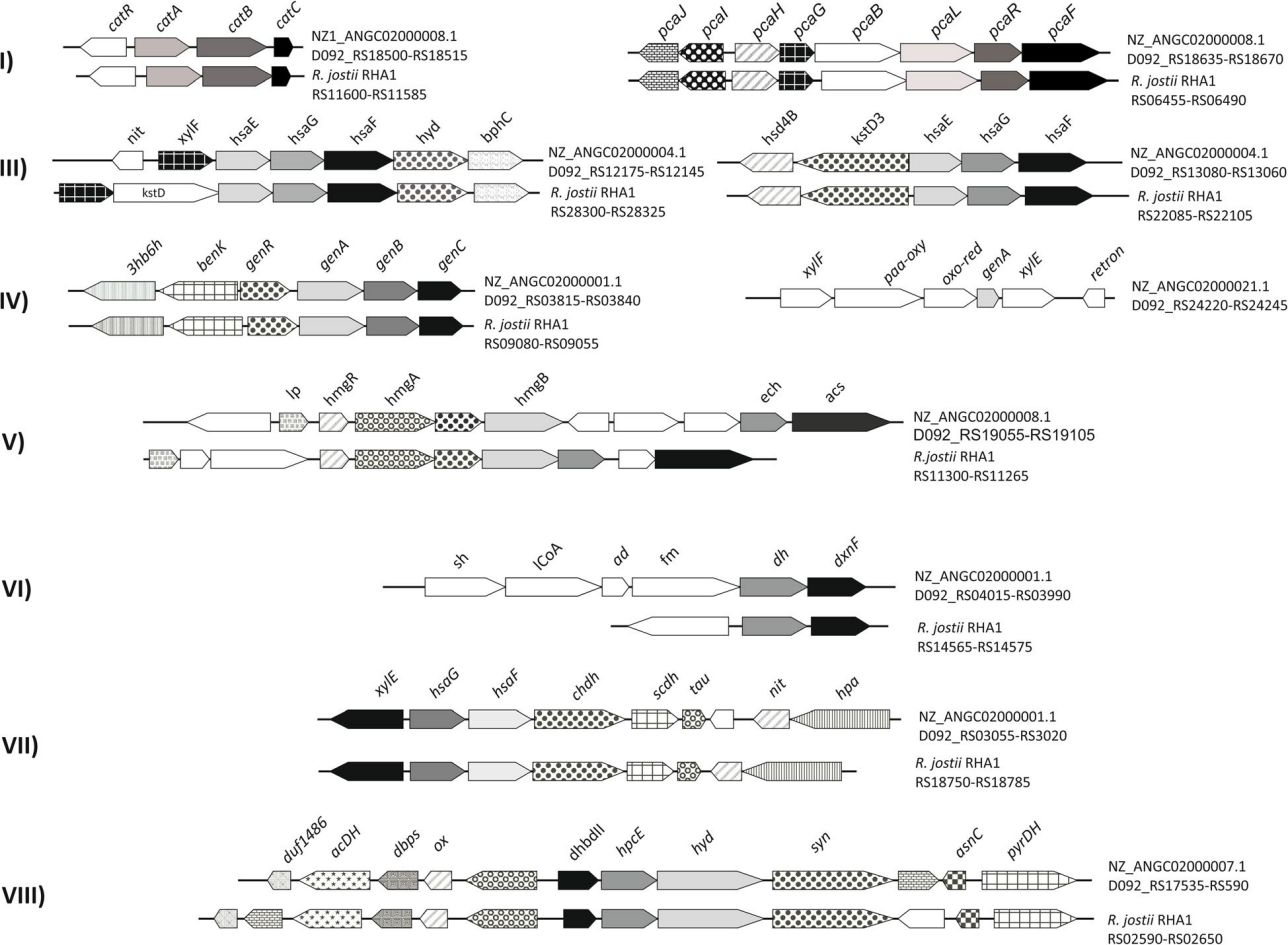
Gene clusters putatively involved in aromatic compounds catabolism identified in *R. ruber* Chol-4 and its comparison with *R. jostii* RHA1. Abbreviations: **I**) ketoadipate pathway: *catR*: transcriptional regulator CatR; *catA*: catechol 1,2 dioyxigenase; *catB*: muconate cycloisomerase; *catC*: mucolactone isomerase; *pcaJ*: succinyl-CoA:3-ketoacid-coenzyme A transferase subunit B; *pcaI*: succinyl-CoA:3-ketoacid-coenzyme A transferase subunit A; *pcaH*: protocatechuate 3,4-dioxygenase β chain; *pcaG*: protocatechuate 3,4-dioxygenase α chain; *pcaB*: 3-carboxy-cis,cis-muconate cycloisomerase; *pcaL*: 4-carboxymuconolactone decarboxylase; *pcaR*: Pca regulon regulatory protein; *pcaF*: β-ketoadipyl-CoA thiolase. **III**) 2-hydroxypentandienoate pathway: *nit*: nitrilotriacetate monooxy7genase component B; *xylF*: 2-hydroxymuconic semialdehyde hydrolase; *hsaE*: 2-hydroxypentadienoate hydratase; *hsaG*: acetaldehyde dehydrogenase, acetylating, it is found in gene cluster for degradation of phenols, cresols, catechol; *hsaF*: 4-hydroxy-2-oxovalerate aldolase; *hyd*: hydroxylase; *bphC*: 2,3-dihydroxybiphenyl 1,2-dioxygenase; *hsd4B*: enoyl-CoA hydratase; *kstD*: 3-ketosteroid-Δ^1^-dehydrogenase. **IV**) gentisate pathway: *3hb6h*:3-hydroxybenzoate 6-hydroxylase; *benK*: benzoate MFS transporter; *genR*: transcriptional regulator (IclR family); *genA*: gentisate 1,2-dioxygenase; *genB*: fumarylpyruvate hydrolase; *genC*: maleylpyruvate isomerase, mycothiol-dependent; *xylF*: 2-hydroxymuconic semialdehyde hydrolase; paa-oxy: 4-hydroxyphenylacetate 3-monooxygenase; *oxo-red*:3-oxoacyl-[acyl-carrier protein] reductase; *xylE*: catechol 2,3-dioxygenase; *retron*: retron-type RNA-directed DNA polymerase. **V**) homogentisate pathway: *lp*: uncharacterized protein Rv2599/MT2674 precursor; lipoprotein; *hmgR*: transcriptional regulator (MarR family); *hmgA*: homogentisate 1,2-dioxygenase; *hmgB*: fumarylacetoacetate hydrolase; *ech*: enoyl-CoA hydratase; *acs:* acetoacetyl-CoA synthetase, long-chain-fatty-acid-CoA ligase. **VI**) hydroxyquinol pathway: *sh*: salicylate hydroxylase; *lCoA*: long chain fatty acid CoA ligase; *ad*: acyl dehydratase; *fm*: FAD-binding monoxigenase; *dh*: iron-containing alcohol dehydrogenase; *dxnF*: hydroxyquinol 1,2-dioxygenase. **VII**) homoprotocatechuate pathway: *xylE, hsaG, hsaF* are previously described; *chdh*: 5-carboxymethyl-2-hydroxymuconate semialdehyde dehydrogenase; *scdh*: putative short chain dehydrogenase; *tau*: 4-oxalocrotonate tautomerase; *nit*: NADH-FMN oxidoreductase-nitrilotriacetate monooxygenase component B; *hpa*: 4-hydroxyphenylacetate 3-monooxygenase. **VIII**) A central pathway with an unknown substrate described in *R. jostii* RHA1: *duf1486:* protein of unknown function DUF1486 (probable NADH dehydrogenase/NAD(P)H nitroreductase); *acDH*: acyl-CoA dehydrogenase, type 2, C-terminal domain; *dbps*:3,4-dihydroxy-2-butanone 4-phosphate synthase /GTP cyclohydrolase II; *ox:* NADH-FMN oxidoreductase; *dhbdII*: biphenyl-2,3-diol-1,2-dioxygenase II (2,3-dihydroxybiphenyl dioxygenase **II**); *hpcE*: possible fumarylacetoacetate hydrolase; *hyd*: FAD-binding monooxygenase (PheA/TfdB family), conserved hypothetical hydroxylase, similar to 2,4-dichlorophenol 6-monooxygenase; *syn*: acetoacetyl-CoA synthetase; *asnC*: transcriptional regulator (AsnC family); *pyrDH*: pyruvate dehydrogenase E1 component. All *R. jostii* RHA1 genes have the prefix “RHA1_” not included in the figure

There are eight central aromatic pathways chromosomally encoded in *R. jostii* RHA1 and *R. opacus* B4 [31, 32] and 7 of them are found in *R. ruber*: 1) the β-ketoadipate or ortho-cleavage pathway encoded by the *pca* and *cat* genes and responsible for the conversion of catechol and protocatechuate into acetyl-CoA and succinyl-CoA by intradiol cleavage of the catecholic intermediate (Fig. 4 I); 2) the phenylacetate pathway encoded by the *paa* genes [33], that takes part in the catabolism of a variety of compounds, including homophthalate, tropate and phenylalkanoates. This pathway was not found in *R. ruber*); 3) the 2-hydroxypentadienoate pathway that transforms 2-hydroxypentadienoates into acetyl-CoA and pyruvate through the successive action of a hydratase, an aldolase and a dehydrogenase (Fig. 4 III) [34]; 4) the gentisate pathway encoded by the *genABC* gene cluster that converts gentisate to pyruvate and fumarate (Fig. 4 IV) [35]; 5) the homogentisate pathway encoded by the *hmgABC* genes that involves the extradiol cleavage of homogentisate, followed by a C-C bond hydrolysis to finally yield fumarate and acetoacetate (Fig. 4 V) [36]; 6) the hydroxyquinol pathway responsible of an intradiol-type cleavage of 4-hydroxysalicylate/hydroxyquinol leading to aceyl-CoA and succinyl-CoA (Fig. 4 VI) [37]; 7) the homoprotocatechuate pathway encoded by the *hpc* genes and involved in the extradiol-type cleavage of homoprotocatechuate (Fig. 4 VII); 8) lastly, a putative metabolic pathway for an unknown substrate that would be made up of a hydroxylase, an extradiol dioxygenase and a hydrolase (Fig. 4 VIII) [31, 32].

### Peripheral pathways

Some of the above aromatic compounds are intermediates in the degradation pathways of other more complex compounds that are also growing substrates of *R. ruber* Chol-4 and whose catabolic pathways meet those previously presented as central pathways. The Chol-4 genes encoding the putative necessary enzymatic activities of these peripheral pathways are described below.

**Fig. 5:**
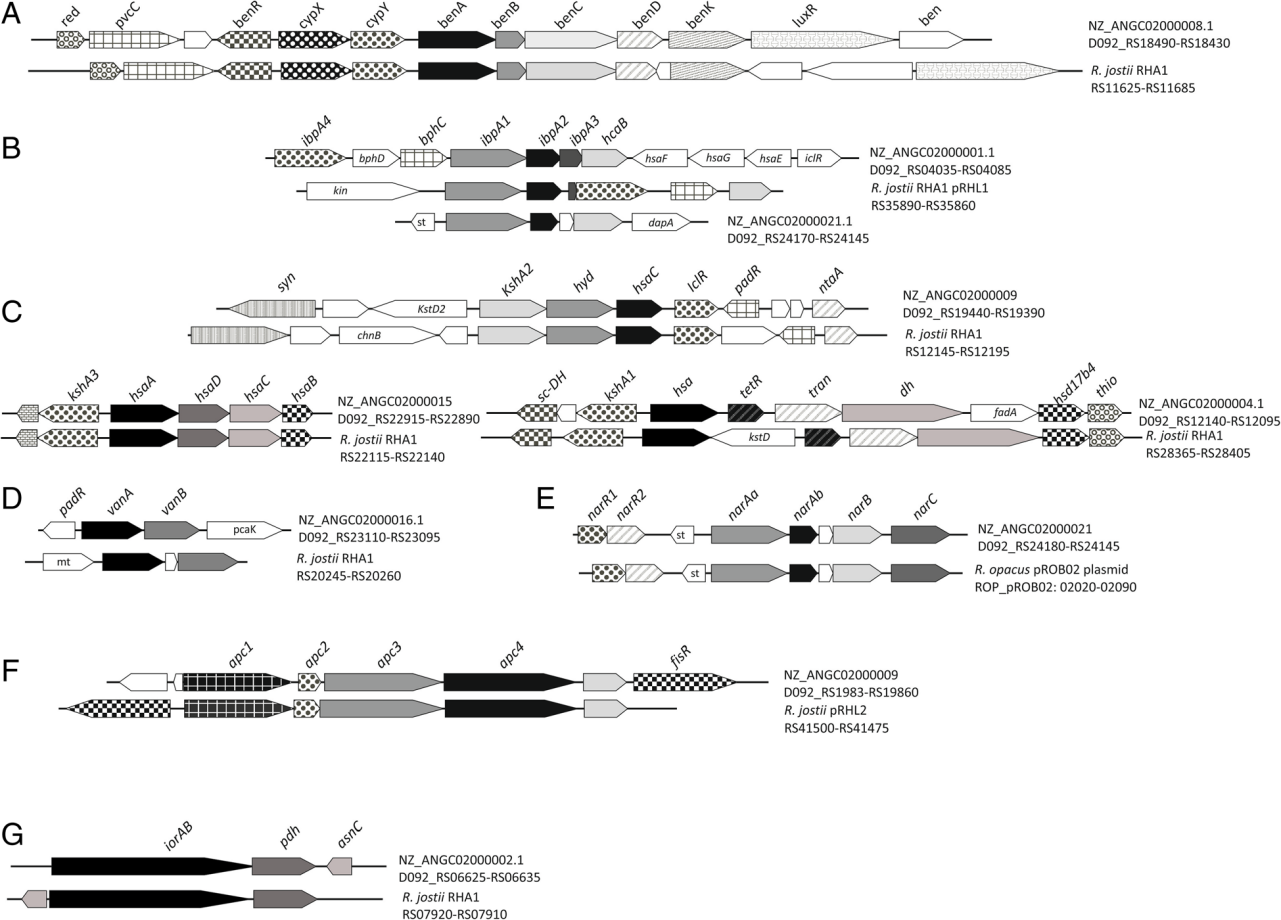
Peripherical routes in *R. ruber*. Abbreviations: **a** Benzoate degradation: *red*: flavin reductase; *pvcC*: pyoverdin chromophore biosynthetic protein; *benR*: transcriptional regulator (AraC family); *cypX*: cytochrome P450 monooxygenase; *cypY*: putative phenol hydroxylase; *benA*: benzoate 1,2-dioxygenase α subunit; *benB*: benzoate 1,2-dioxygenase β subunit; *benC*: benzoate dioxygenase, ferredoxin reductase component/1,2-dihydroxycyclohexa-3,5-diene-1-carboxylate dehydrogenase; *benD*: 1,2-dihydroxycyclohexa-3,5-diene-1-carboxylate dehydrogenase; *benK*: benzoate MFS transporter; *luxR*: transcriptional regulator (luxR family) putative.; *ben*: benzoate transport protein. **b** Isopropylbenzene pathway: *ipbA4*: ferredoxin reductase; *bphD:* 2-hydroxy-6-oxo-2,4-heptadienoate hydrolase; *bphC*: 2,3-dihydroxybiphenyl 1,2-dioxygenase; *ipbA1*: isopropylbenzene 2,3-dioxygenase or IPB-dioxygenase, ISP large subunit; *ipbA2*: IPB-dioxygenase (ISP small subunit); *ipbA3*: IPB-dioxygenase ferredoxin; *hcaB*: hydroxybenzaldehyde dehydrogenase*; hsaF, hsaG; hsaE*: previously described (Fig. 4); *iclR*: transcriptional regulator (IclR family); *kin*: sensor kinase; *st*: sterol-binding domain protein; *dapA*: 4-hydroxy-tetrahydrodipicolinate synthase. **c** Steroids pathway: *syn*: non-ribosomal peptide synthetase; *kstD*: 3-oxosteroid 1-dehydrogenase; *kshA*: ketosteroid-9-α-hydroxylase, oxygenase; *hyd*: hydroxylase; *hsaC*: 2,3-dihydroxybiphenyl 1,2-dioxygenase; *iclR*: transcriptional regulator (IclR family); *padR*: transcriptional regulator (PadR family); *ntaA*: nitrilotriacetate monooxygenase component B; *chnB*: cyclohexanone monooxygenase; *hsaA*: flavin-dependent monooxygenase; *hsaD*: 2-hydroxy-6-oxo-6-phenylhexa-2,4-dienoate hydrolase; *hsaC*: iron-dependent extradiol dioxygenase; *hsaB*: flavin-dependent monooxygenase reductase subunit; *sc-DH*: short-chain dehydrogenase; *hsa*: monooxygenase; *tetR*: probable transcriptional regulator (TetR family); *tran*: acetyl-CoA acetyltransferase; *dh*: acyl-CoA dehydrogenase; *fadA*: 3-ketoacyl-CoA thiolase; *hsd17b4*: 3-α,7-α,12-α-trihydroxy-5-β-cholest-24-enoyl-CoA hydratase; *thio*: thioesterase. **d** Vanillate: *padR*: transcriptional regulator (PadR family); *vanA*: vanillate *o*-demethylase oxygenase subunit, flavodoxin reductases (ferredoxin-NADPH reductases) family 1; *vanB*: vanillate *o*-demethylase oxidoreductase; *pcaK*: 4-hydroxybenzoate transporter; mt:methyltransferase. **e** Naphtalen. (*nar* genes*: R. opacus* plasmid pROB02:AP011117): *narR1* and *narR2*: putative naphthalene degradation regulatory protein; *narAa*: nidA, naphthalene dioxygenase large subunit; *narAb*: (nidB) naphthalene dioxygenase small subunit; *narB*: (nidC) 1,2-dihydro-1,2-dihydroxynaphthalene dehydrogenase; *narC*: (nidD) putative aldolase NarC. **f** Acetophenone carboxylase (anaerobic): *apc1–4*: acetophenone carboxylase subunits; *fisR*: transcriptional regulato (Fis family). **g** Aminoacid: *iorAB*: indolepyruvate ferredoxin oxidoreductase (α and β subunits); *pdh*: glutamate / leucine / phenylalanine / valine dehydrogenase; *asnC*: transcriptional regulator (AsnC family). All *R. jostii* RHA1 genes have the prefix “RHA1_” not included in the figure

**Fig. 6:**
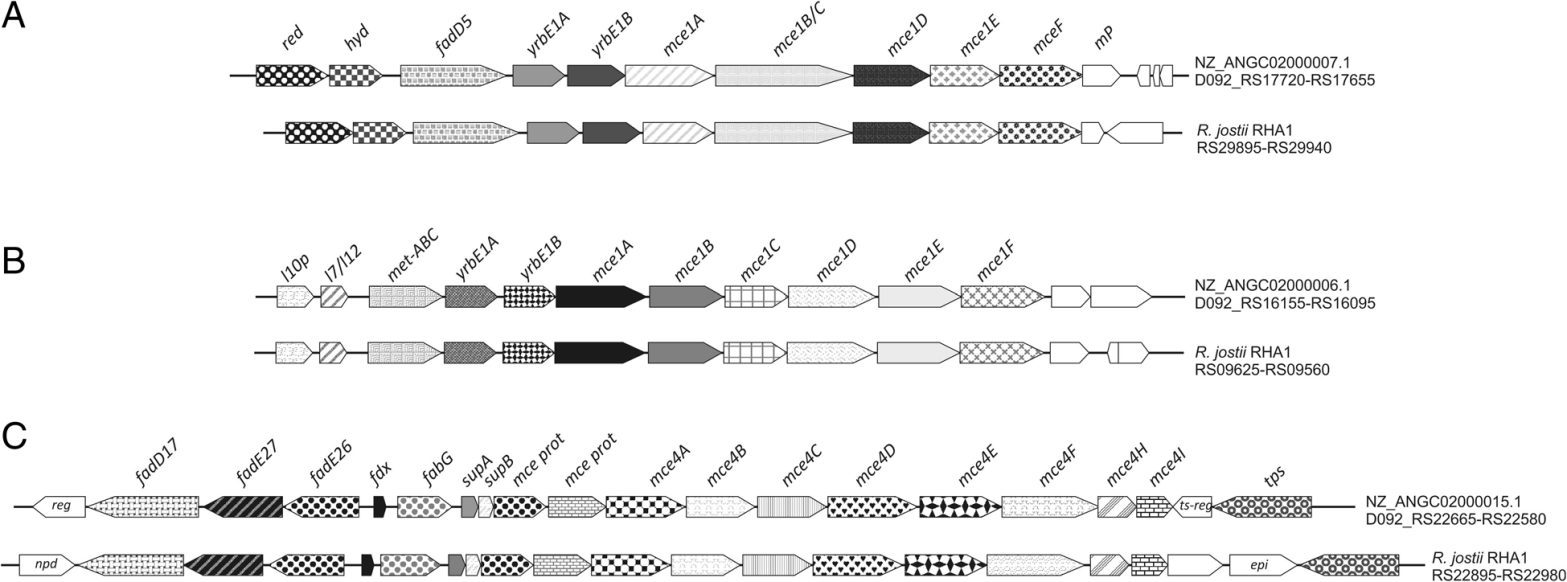
*Mce* systems in *R. ruber*. Abbreviations: **a** *red*: enoyl-[acyl-carrier-protein] reductase [FMN]; *hyd*: enoyl-CoA hydratase; *fadD5*: long-chain fatty-acid-CoA ligase, *Mycobacterial* subgroup FadD5; *yrbE1A*: conserved hypothetical integral membrane protein YrbE1A (ABC-transporter permease); *yrbE1B*: conserved hypothetical integral membrane protein YrbE1B (ABC-transporter permease); *mce1A-D*: MCE-family protein MceA-D; *mce1E*: MCE-family lipoprotein LprK (MCE-family lipoprotein Mce1e); *mceF*: MCE family protein of Mce F Subgroup; *mp*: membrane protein. **b** *l10p*: LSU ribosomal protein L10p (P0); *l7/l12*: LSU ribosomal protein L7/L12 (P1/P2); *met-ABC*: methionine ABC-transporter ATP-binding protein (*npd*:2-nitropropane dioxygenase, NPD). **c** *reg*: possible transcriptional regulatory protein; *fadD17*: long-chain fatty-acid-CoA ligase (*Mycobacterial* subgroup FadD17); *fadE27*: butyryl-CoA dehydrogenase; *fadE26*: acyl-CoA dehydrogenase (*Mycobacterial* subgroup FadE26); *fdx*: ferredoxin; *fabG*: 3-ketoacyl-ACP reductase (hsd4A); *supA* and *supB*: ABC-transporter permease; *ts-reg:* two-component system response regulator; *tps*: α,α-trehalose-phosphate synthase [UDP-forming]; *npd*: acyl-CoA synthetase; *epi*: epimerase, dihydroflavonol-4-reductase. All *R. jostii* RHA1 genes have the prefix “RHA1_” not included in the figure

Gene clusters *ben*, *cat* and *pca* (Fig. 4i and Fig. 5a) could be involved in the benzoate degradation. Isopropylbenzene degradation genes were found in a gene cluster in NZ_ANGC02000001.1 contig, and also in a different gene cluster in NZ_ANGC02000021.1 contig (Fig. 5b). In Fig. 5c some of the steroid catabolic gene clusters contained in the *R. ruber* genome are depicted. Transport systems related to steroid molecules are also of interest and therefore mammalian cell entry (MCE) systems that have been associated with steroid transport [38, 39] were searched through the genomic data. Figure 6 collects all the MCE systems found in the *R. ruber* genome.

A putative gene cluster for vanillate degradation was found in *R. ruber* genome (GenBank: Y11521, Fig. 5d). Probably involved in naphtalene catabolism, a *nar* gene cluster containing two regulatory genes, *narR1* belonging to the GntR family and NarR2 of the NtrC family of enhancer-binding proteins has been also found in the *R. ruber* Chol-4 genome (Fig. 5e). NZ_ANGC02000009.1 contig (Fig. 5f) contained a putative anaerobic degradation gene cluster for an acetophenone carboxylase activity. There was also another cluster containing a phenylalanine dehydrogenase putatively involved in amino acid catabolism (Fig. 5g).

Other gene clusters that occur in related bacteria were not found in the *R. ruber* Chol-4 genome, such as the *bph* cluster for biphenyl catabolism, [40] and the *pad* cluster for phthalate catabolism [41].

### Growth in different organic compounds

In order to determine the growth capabilities of *Rhodococcus ruber* strain Chol-4, we analyzed its ability to use several compounds as sole energy and carbon source (Table 1).

**Table 1:** Growth of *R. ruber* Chol-4 wild type on minimum medium with different carbon sources

Aromatic compounds	Growth	Volatile compounds	Growth
catechol	+	13-butanediol	+
cholic acid	+	2,3-butanediol	+
DHEA	+	Isopropanol	+
gentisate	+	benzene	–
homogentisate	+	ethylbenzene	–
L-tryptophan	+	indane	–
naphthalene	+	phenylacetic acid	–
phenol	+	styrene	–
protocathecuate	+	tetralin	–
sodium benzoate	+	toluene	–
vanillic acid	+	xylene isomers	–
2-aminobenzoate	–		
biphenyl	–		
hydroxyquinol	–		
L-tyrosine	–		
phthalate	–		
salicylic acid	–		

**Fig. 7:**
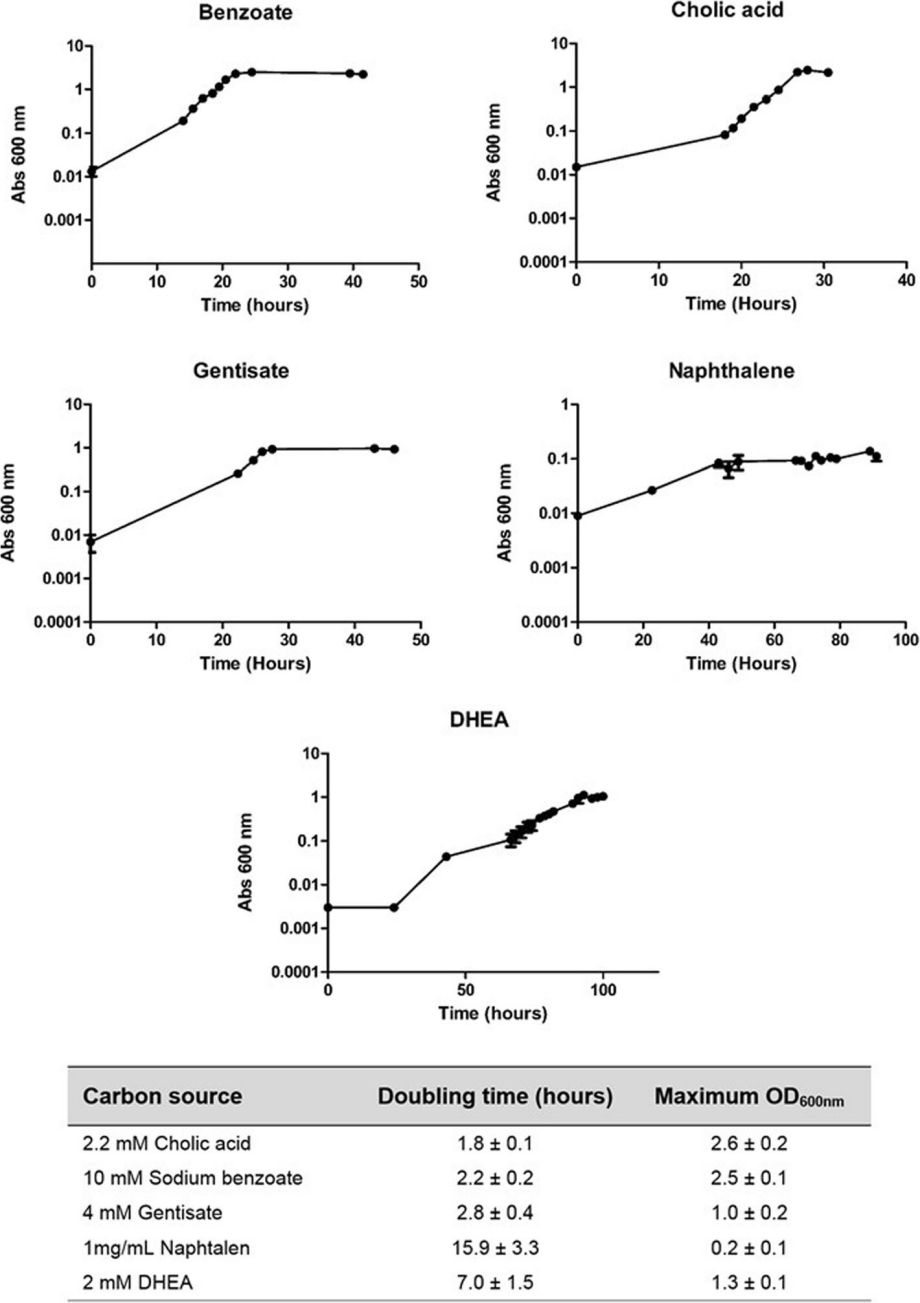
Growth curves of *R. ruber* WT in minimal medium 457 supplemented with cholic acid, sodium benzoate, gentisate or DHEA. In the case of cholic acid and DHEA 16.5 mM cyclodextrins were present in the medium to increase the solubility of the compounds (no growth when using only cyclodextrins was observed). *R. ruber* was also grown in minimal medium 457 supplemented with naphthalene in powder (1 mg/mL). Data of 3–4 independent experiments are depicted. The standard error of the mean was calculated by GraphPad Prism 5.0

The growth curves of *R. ruber* with some of the metabolizable compounds (sodium benzoate, cholic acid, gentisate, naphthalene or DHEA as sole carbon source) are shown in Fig. 7.

**Fig. 8:**
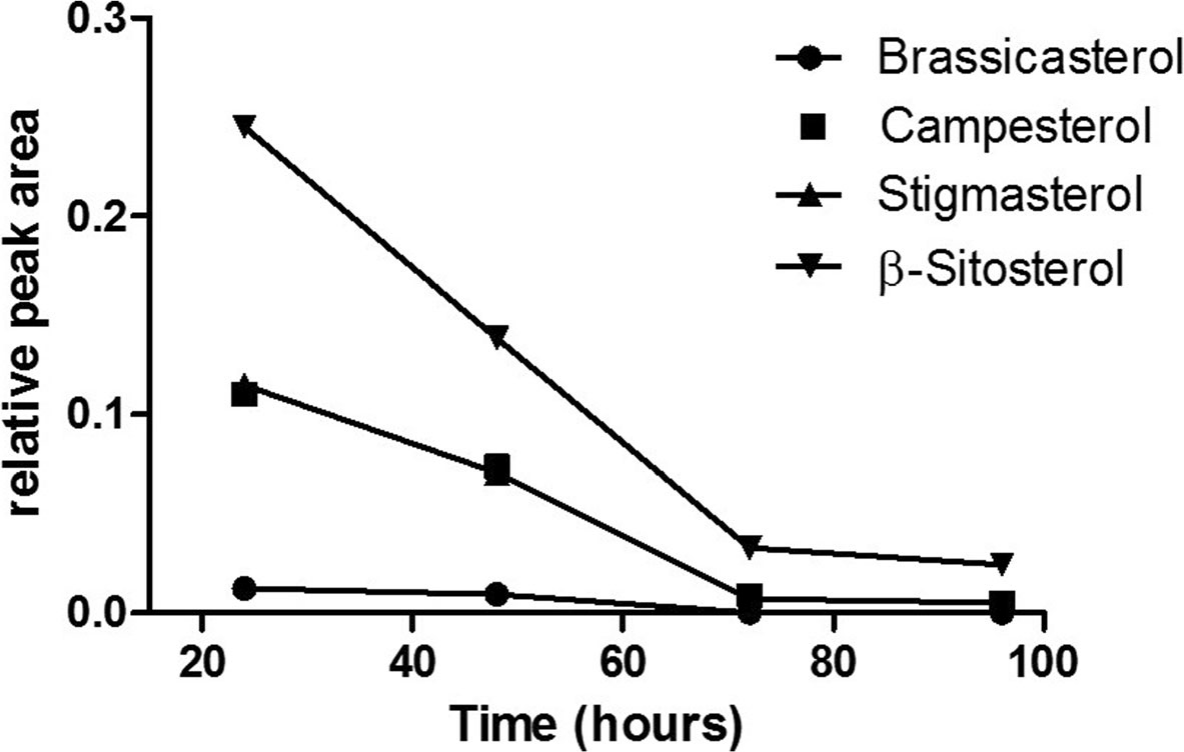
*R. ruber* phytosterols comsumption. The strain was inoculated in minimal medium 457 supplemented with a mixture of phytosterols added in powder (brassicasterol, campesterol, stigmasterol and β-sitosterol). The panel shows the consumption of each compound (y-axis, relative peak area) versus the time since the phytosterol addition to the medium (x-axis). The relative peak area is the ratio between the HPLC peak obtained for each phytosterol and the peak of pregnenolone used as internal control. A representative experiment is shown

*R. ruber* was isolated by its capacity to degrade steroid compounds like cholesterol. In this work, its growth capabilities using other steroids of interest such as plant sterols was investigated. Cells were grown on minimal medium supplemented with a mix of phytosterols (plant sterols that included brassicasterol, campesterol, stigmasterol and β-sitosterol) as only source of energy and carbon. The sterol consumption was followed by HPLC. The results proved that sterol concentration was reduced to 5% of the initial value (Fig. 8).

### Mutant construction

**Fig. 9:**
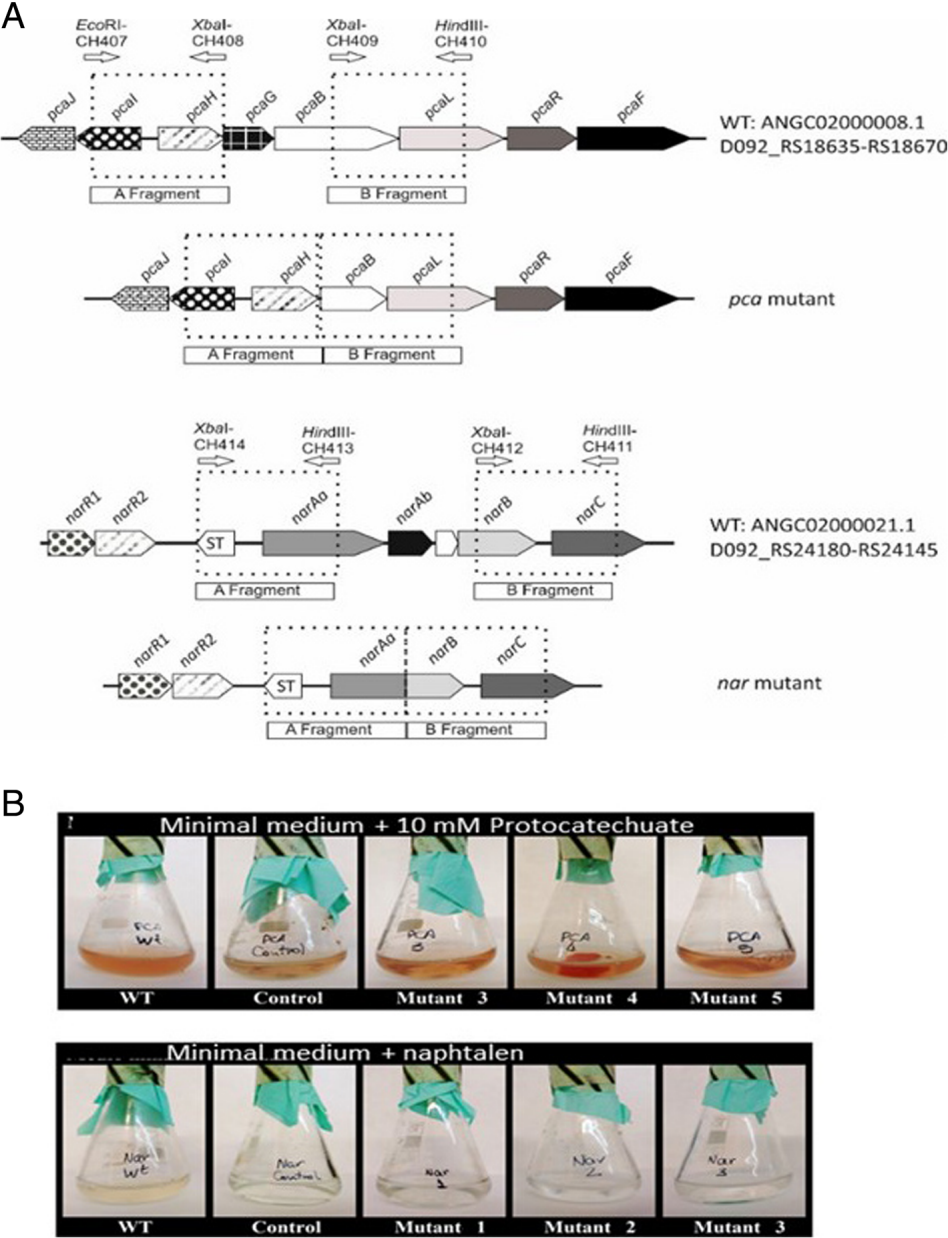
*Rhodococcus ruber* mutants. **a** Scheme of *R. ruber* deletion *pca* mutant and *nar* mutant**. b** Growth of *R. ruber* on minimal medium supplemented with 10 mM PCA or naphthalene in powder (1 mg/mL), respectively. WT: wild type; *pca* mutant (3, 4 and 5) and *nar* mutant (1–3); control: non-existent growth in the absence of inoculum

Unmarked gene deletions were carried out in the *pca* and *nar* gene clusters of *R. ruber* strain Chol-4 to verify the involvement of these genes in the growth of protocatechuate and naphthalene, respectively. A scheme of the introduced deletions is depicted in Fig. 9 A. Mutants were confirmed by PCR and growth experiments proved that *nar R. ruber* mutants lost the ability to grow on naphthalene; similarly, *pca R. ruber* mutants were not able to grow on protocatechuate (Fig. 9 B).

On the other hand, the growth of the *nar* and *pca R. ruber* mutants was also checked with different carbon sources (Table 2). The *nar* mutant could grow in all alternative substrates tested. The *pca* mutant, however, lost the capability to grow in vanillate.

**Table 2:** Growth of *R. ruber* Chol-4 mutants on minimum medium with different carbon sources

Strain/carbon source	Naph	Benz	Tryp	Cat	Homog	Gen	PCA	Chol	Vanill
Wild type	+	+	+	+	+	+	+	+	+
*nar* mutant	–	+	+	+	+	+	+	+	+
*pca* mutant	+	+	+	+	+	+	–	+	–

*Naph* Naphthalen 7 mM, *Benz* Benzoate 10 mM, *L-Tryp* L-tryptophan 5 mM, *Cat* Catechol 10 mM, *Homog* Homogentisate 2 mM, *Gen* Gentisate 4 mM, *PCA* Protocatechuic acid 10 mM, *Chol* Cholic acid 2.2 mM, *Vanill* Vanillic acid 4 mM

## Discussion

### General genome features

The sequence data employed in this study is the integrated results of two independent sequencing experiments (NCIB database: NZ_ANGC00000000.2). In this work, we present a more comprehensive genomic analysis on this revised sequence, and a comparative analysis with other already described *Rhodococcus* genome sequences. The published genome size of different *Rhodococcus* is in the range of 3.9 to 10 Mb. This large difference in genome size could be related both to the presence of large plasmids and to the extensive genome instability that occurs in several *Rhodococcus* species [42]. The genome of *R. ruber* Chol-4 is 5.4 Mb long, a size close to *Rhodococcus* genome average size (5.69 Mb: https://www.ncbi.nlm.nih.gov/genome/13525) and quite similar to the *R. equi* ATCC 33707 (5.2 MB), *R. pyridinivorans* SB3094 (5.6 Mb), *R. aetherivorans* or *R. fascians* A44A (both with 5.9 Mb) genome.

The analysis of the genomic data suggests that *R. ruber* Chol-4 contains several putative metabolic gene clusters of biotechnological interest, particularly those involved in the catabolism of aromatic compounds (clusters related to the metabolism of benzoate, vanillate, naphtalen, gentisate, etc.) and steroids (for instance, clusters related to cholesterol catabolism) supporting its potential as a model organism for studying aromatic molecules and steroid biodegradation.

Rhodococci are very interesting microorganisms because of their ability to degrade a broad spectrum of aromatic molecules, which are structures very difficult to catabolize and widely distributed in the biosphere. In *Rhodococcus* strains, the catabolism of aromatic compounds is organized in a modular way that includes peripheral, central and basic pathways (Fig. 3). In the peripheral pathways, aromatic compounds (e.g. biphenyl and phthalate) are converted into specific intermediates (e.g. catechol and phenylacetate) that, in turn, are used in central aromatic pathways to produce a set of common intermediates (e.g. tricarboxylic acid cycle metabolites) that finally are substrates for the basic pathways (Fig. 3) [32]. This kind of organization has been previously named catabolon in other organisms, such as *Pseudomonas* [43].

The central aromatic pathways constitute a catabolic core present in most rhodococci. *R. ruber* Chol-4 genome contains 7 out of the 8 central aromatic pathways described in both *R. jostii* RHA1 and *R. opacus* B4 [31, 32] (see Fig. 3a) being absent only the phenylacetate pathway. This pathway seems to be characteristic of larger genomes as most of the smallest *Rhodococcus* genomes (*R. equi*, *R. aetherivorans* and *R. pyridinivorans* among them) lack also the phenylacetate pathway. However, other pathways such as the genes encoding the gentisate, the homoprotocatechuate and the named VIII pathways found in RHA1 are absent in at least two *R. erythropolis* strains: PR4 and SK121 [32]. Therefore, although most of the aromatic central pathways are conserved within rhodococci, there are metabolic differences among species that could be related to their genome size.

On the other hand, some of the genes detected in the central pathways are redundant in the genome of *R. ruber* Chol-4. Apart from certain enzymatic activities such as KstD and Ksh isoforms previously described in this strain within the steroids catabolism [19, 44, 45],there are also redundant clusters in the *R. ruber* genome such as 3 copies of *pcaJ-pcaI* involved in the catechol and protocatechuate pathways of the β-ketoadipate and leucine catabolism that encodes a succinyl-CoA: 3-ketoacid-coenzyme A transferase (EC 2.8.3.5) in NZ_ANGC02000003.1 (D092_RS10690-RS10695), NZ_ANGC02000026.1 (D092_RS24595-RS24600) and NZ_ANGC02000008.1 (D092_RS18665-RS18670) contigs with an amino acid identity of 66–73% among them and the 2 copies of the *hsaEGF* cluster (NZ_ANGC02000004.1 contig; Fig. 4 III) with an amino acid identity of 65–78% belonging to the 2-hydroxypentanodienoate pathway. Gene redundancy in Rhodocci in both catabolic and anabolic pathways is proposed to facilitate high metabolic versatility [44–46] or as a mechanism to increase their potential to adapt to new carbon sources [41].

More complex aromatic compounds are partially degraded in the peripheral pathways until they reach one of the intermediates that are substrates of the central aromatic pathways. The number of peripheral pathways present in every Rhodococcus species is variable, probably related with the genomic size or the plasmid content.

In *Rhodococcus ruber* the pathways related to the catabolism of benzoate, isopropylbenzene, vanillate, napthalen and steroids, among others has been identified. The benzoate clusters *ben*, *cat* and *pca*, are present in the *R. ruber* Chol-4 genome closely located and organized in a similar way to that in *R. jostii* RHA1 (Figs. 4 and 5a) [41]. *Rhodococcus jostii* strain RHA1 catabolizes benzoate via the cathecol pathway that includes a ring-hydroxylating oxygenase [41]. The cathecol and the protocatechuate branches of the β-ketoadipate pathway converge at the β-ketoadipate enol-lactone in this strain.

In *R. ruber*, the genes encoding the isopropylbenzene catabolic pathway are located in NZ_ANGC02000001.1 and NZ_ANGC02000021.1 contigs (Fig. 5b). This aromatic hydrocarbon compound is a constituent in crude oil and refined fuels. The isopropylbenzene gene cluster *ipbA1A2A3A4C* codes for a reductase (*ipbA4*), a ferredoxin (*ipbA3*), a dioxygenase (*ipbA1A2*) and a 3-isopropylcatechol-2,3-dioxygenase (*ipbC*) [47].

A gene cluster for vanillate catabolism is found in the *R. ruber* Chol-4 genome (GenBank: Y11521, Fig. 5d) and it is similar to the gene loci *vanA* and *vanB* of *Pseudomonas* sp. strain HR199. Vanillate is a lignin-derived methoxylated monocyclic aromatic compound whose catabolism proceeds via protocatechuate in *Comamonas testosteroni* strain BR6020 and in *Pseudomonas* sp. strain HR199 [48, 49].

The naphtalene-involved *nar* gene cluster found in *R. ruber* Chol-4 (Fig. 5e) is similar to the cluster present in the plasmid pROB02 of *Rhodococcus opacus* B4 (NC_012521). In *Rhodococcus* sp. strain NCIMB 12038 and *Rhodococcus opacus* R7 the activities proposed to be encoded in the *nar* cluster are: i) a gentisate 1,2-dioxygenase that converts gentisate into maleylpyruvate; ii) a mycothiol-dependent maleylpyruvate isomerase that catalyzes the isomerization of maleylpyruvate to fumarylpyruvate; and iii) a fumarylpyruvate hydrolase that hydrolyzes fumarylpyruvate to yield fumarate and pyruvate [50, 51]. The gentisate degradation pathway is shared by both the naphthalene and the 3-hydroxybenzoate catabolism. The *nar* gene cluster presents a diverse genetic organization with different kind of regulators among *Rhodococcus* strains [51].Among the genes involved in the last pathway, the dioxygenase *thnA1234* cluster could correspond to the isopropylbenzene *ipb1234* cluster found in the *R. ruber* genome (Fig. 5b). Therefore, naphthalene could be catabolized in *R. ruber* via either the *nar* genes or the isopropylbenzene cluster.

*Rhodococcus ruber* contains many related-steroid clusters (Fig. 5c and Fig. 6). We previously reported other steroid clusters, conferring the ability to grow in different steroids (such as cholesterol, cholestenone, testosterone, 1,4-adrostadien-3,17-dione or 4-7adrostene-3,17-dione), and the role of some enzymes such as ketosteroid dehydrogenases, ketosteroid 9-α hydroxylases and cholesterol oxidase [14, 19, 44, 45, 52].

Rhodococci are so broadly known as competent steroid degraders [11] that they could be considered as the steroid-consumer strains by excellence. The cholesterol catabolic pathway has been widely studied, revealing a notable complexity in part due to the existence of alternative pathways and the diversity of the enzymes involved. As steroid intermediates are highly appreciated in the pharmaceutical industries, the steroid catabolic capacity of *R. ruber* strain Chol-4 represents a promising biotechnological platform for the production of steroid drugs.

The steroid degradation genes are generally organized within large gene clusters [53] and this seems also to be the case in *R. ruber* Chol-4. For instance, the cholate catabolic gene cluster found in RHA1 [54] is also present in the *R. ruber* Chol-4 genome (Fig. 5c). Other steroid genes, encompassing the MCE systems, are involved in steroid transport in actinobacteria. Every MCE system is an ATP-binding cassette transporter comprising more than eight distinct proteins. The number of MCE systems could vary among bacteria: from 4 in *Mycobacterium tuberculosis* H37Rv to 6 in *M. smegmatis*. The MCE4 system of *Rhodococcus jostii* RHA1 or *Mycobacterium smegmatis* has been proved to be an active uptake system that requires ATP to transport steroids such as cholesterol, 5-α-cholestanol, 5-α-cholestanone or β-sitosterol [38, 39]. The other *mce* operons could be involved in the cell envelope structure maintenance [39]. We found three MCE systems in *R. ruber* Chol-4 (Fig. 6). One of them, lying in NZ_ANGC02000015.1 contig, exhibited the higher similarity with the *mce4* system of RHA1. Consequently, we propose that this MCE system would be related to steroid transport.

On the other hand, although *R. ruber* can grow on cholate [44], no ORFs similar to the RHA1 cholate transport system, i.e. the ABC-transporter *CamABCD* ro04888 to ro04885 and *CamM* ro05792 [55] were detected. This suggest that cholate transport systems could differ within *Rhodoccus* species.

### Experimental analysis of *R. ruber* catabolic capabilities

The growth results were in accordance with the theoretical data from the identification of gene clusters within the genome of *Rhodococcus ruber* Chol-4. For instance, the failure to grow on volatile compounds (benzene, toluene, etc.) could be explained by the absence of specific clusters involved in the catabolism of these compounds.

However, there were some interesting exceptions: *R. ruber* Chol-4 did not grow on hydroxyquinol despite the fact that the pathway VI genes are present in its genome (see Fig. 3). It neither grew on salicylate, although up to two putative salicylate hydroxylases (EC 1.14.13.1) were found in its genome (D092_RS04015 in NZ_ANGC02000001.1 contig and D092_RS16585 NZ_ANGC02000006.1 contig).

Some interesting observations were revealed by the in vitro growth experiments. *R. ruber* grew on benzoate, catechol and protocatechuic acid. Therefore, the catabolism of benzoate could take place via the cathecol pathway through a ring-hydroxylating oxygenase as it has been proposed for RHA1 [41]. On the other hand, *R. ruber* grew on naphthalene as sole organic substrate (Fig. 7 and Table 1). As stated before, two different pathways for naphthalene catabolism have been described in *Rhodococci* to date, one relying on the *nar* cluster and the other relying on the isopropylbenzene cluster (*ipb*) [51, 56], both converging on salicylate which is subsequently hydroxylated to gentisate. Thus, the fact that *R. ruber* grew in naphthalene and gentisate, but not in salicylate, was perplexing, and suggested that the intake of this compound might be hampered or, a more provoking hypothesis, that this strain catabolizes naphthalene through an alternative pathway that would not involve salicylate as intermediate. More studies should be taken to elucidate this apparent paradox.

### Mutant construction

In order to check the functionality of several of the pathways putatively identified in *R. ruber* two groups of genes were deleted. On one hand, protocatechuate 3,4-dioxygenase α chain (*pcaG*) and the 3-carboxy-cis,cis-muconate cycloisomerase (*pcaB*) genes of the cluster related to the protocatechuic acid pathway were deleted (Figs. 9 and 4I). Th*e R. ruber* Chol-4 *pca* mutants were not able to grow on protocatechuate (Fig. 9) showing that the *pca* gene cluster is directly involved in the metabolism of protocatechuate. The growth on vanillate also resulted to be dependent on the *pca* cluster in this strain (Fig. 3). A RHA1 *pc*a mutant also failed to grow on vanillate as the sole organic substrate suggesting that this substrate is degraded via the β-ketoadipate pathway [57]. On the other hand, deletion of the naphthalene dioxygenase *nar* genes led to the loss of growth on naphthalene (Figs. 9 and 5e). Therefore, the *nar* gene cluster is responsible of the naphthalene catabolism in *R. ruber* Chol-4, while the *ipb* gene cluster is not involved in that degradation.

## Conclusions

In summary, the analysis of the *Rhodococcus ruber* strain Chol-4 genome substantiated its relevance as a model organism for studying steroid and aromatic compounds biodegradation. The agreement between gene clusters found in the genome and the growth results of *R. ruber* has been established. *R. ruber* is able to grow in minimal medium with steroids (e.g. cholesterol, phytosterols, DHEA), bile acids (cholic acid) or several aromatic compounds (e.g. benzoate, naphthalene, gentisate) as the only source of carbon and energy. Deeper studies on Chol-4 degradation capabilities based on the construction of some mutants revealed that the *nar* gene cluster is indeed involved in the naphthalene catabolism in *R. ruber*, while the *pca* gene cluster is responsible of the metabolism of both protocatechuate and vanillate.

Our results confirm and reinforce the biotechnological interest of *R. ruber* strain Chol-4 due to its metabolic potential that opens a great variety of applications as, for instance, its use in the bacterial transformation of steroids to produce pharmaceutically active steroid drugs. Further studies will be focused in exploring *R. ruber* Chol-4 novel potential biotechnological applications.

## Additional files

Additional file 1: **Table S1.** Bacterial strains and plasmids used in this work. (DOCX 17 kb)

Additional file 2: Growth in gas phase via saturated atmosphere. (JPG 88 kb)

Additional file 3: **Table S2.** Primers used in this work. (DOCX 13 kb)

Additional file 4: **Figure S1.** Sequence length vs GC content of the 129 scaffolds obtained in the initial assembly of *R. ruber* Chol-4 genome. (DOCX 428 kb)

Additional file 5: Quast genome assembly evaluation. (PDF 30 kb)

Additional file 6: **Table S3.** Anticodons encoded in the *R. ruber* Chol-4 genome. (DOCX 20 kb)

Additional file 7: **Table S4.** List of mobile elements found in the *R. ruber* Chol-4 genome. (DOCX 25 kb)

Additional file 8: **Table S5.** List of recombinases identified in the *R. ruber* Chol-4 genome. (DOCX 19 kb)

Additional file 9: **Table S6.** List of restriction modification systems identified in the *R. ruber* Chol-4 genome. (DOCX 18 kb)

## Abbreviations

CRISPR: Clustered regularly interspaced short palindromic repeats
DHEA: Dehydroepiandrosterone
HPLC: High performance liquid chromatography
LB: Luria Bertani
MCE: Mammalian cell entry system
NGS: Next Generation Sequencing
ORF: Open reading frame
PCR: Polymerase chain reaction
