# Supplementary material for: New insights into the genome of Rhodococcus ruber strain Chol-4
Source: BMC Genomics. 2019 May 2;20:332. doi: 10.1186/s12864-019-5677-2 (PMC6498646; doi:10.1186/s12864-019-5677-2)
Supplement: Supplementary file 1 — Table S1. Bacterial strains and plasmids used in this work. (DOCX 17 kb) [file 12864_2019_5677_MOESM1_ESM.docx]

**Additional file 1: Table S1**. Bacterial strains and plasmids used in this work.

| Strain and plasmids | Description | Reference |
| --- | --- | --- |
| *E. coli* DH5α | F’ *endA1 hsdR17* (r_K_^-^ m_K_^+^) *glnV44 thi-1 recA1 gyrA* (Nal^R^) *relA1* Δ(*lacIZYA-argF*) *U169* *deoR* (φ80*dlac*Δ(*lacZ*)*M15*). | Laboratory collection |
| *E. coli* GM48 | F^–^ thr leu thi lacY galK galT ara fhuA tsx dam dcm glnV44 | CGSC5127 |
| *E. coli* S17.1 | *recA pro hsdR RP4-2-Tc::Mu-Km::Tn7*. | [1] |
| *Rhodococcus ruber* strain *Chol-4* | Wild type phenotype, Nal^R^ | [2] |
| *nar* mutant | *Rhodococcus ruber* Chol-4 mutant in *nar* genes, Nal^R^. | This work |
| *pca* mutant | *Rhodococcus ruber* Chol-4 mutant in *pca* genes, Nal^R^. | This work |
| pGem-T Easy | Cloning vector *E. coli*, Ap^R^ | Promega |
| pK18mobsacB | Km^R^, RP4mob,mobilizable cloning vector with *sacB* gene for positive selection | [3] |
| pK18A | pK18mobsacB with fragment A from the *nar cluster* cloned between *Eco*RI and *Xba*I. | This work |
| pK18AB | pK18mobsacB with fragments A and B from the *cluster* *nar* cloned between *Eco*RI and *Hind*III. | This work |
| pK18C | pK18mobsacB with the fragment C from the *pca* *cluster* cloned between *Eco*RI and *Xba*I. | This work |
| pK18CD | pK18mobsacB with fragments C and D from the *pca* *cluster* cloned between *Eco*RI and *Hind*III. | This work |

1. Simon R, Priefer U, Pühler A. A broad host range mobilization system for in vivo genetic engineering: transposon mutagenesis in gram negative bacteria. Biotechnology 1983; 1:748-791.

2. Fernández de las Heras L, García Fernández E, María Navarro Llorens J, Perera J, Drzyzga O. Morphological, physiological, and molecular characterization of a newly isolated steroid-degrading actinomycete, identified as *Rhodococcus ruber* strain Chol-4. Curr Microbiol 2009; 59:548-553.

3. Schafer A, Tauch A, Jager W, Kalinowski J, Thierbach G, Puhler A. Small mobilizable multi-purpose cloning vectors derived from the *Escherichia coli* plasmids pK18 and pK19: selection of defined deletions in the chromosome of *Corynebacterium glutamicum*. Gene 1994; 145:69-73.
