# Supplementary figures and images for: New insights into the genome of Rhodococcus ruber strain Chol-4
Source: BMC Genomics. 2019 May 2;20:332. doi: 10.1186/s12864-019-5677-2 (PMC6498646; doi:10.1186/s12864-019-5677-2)

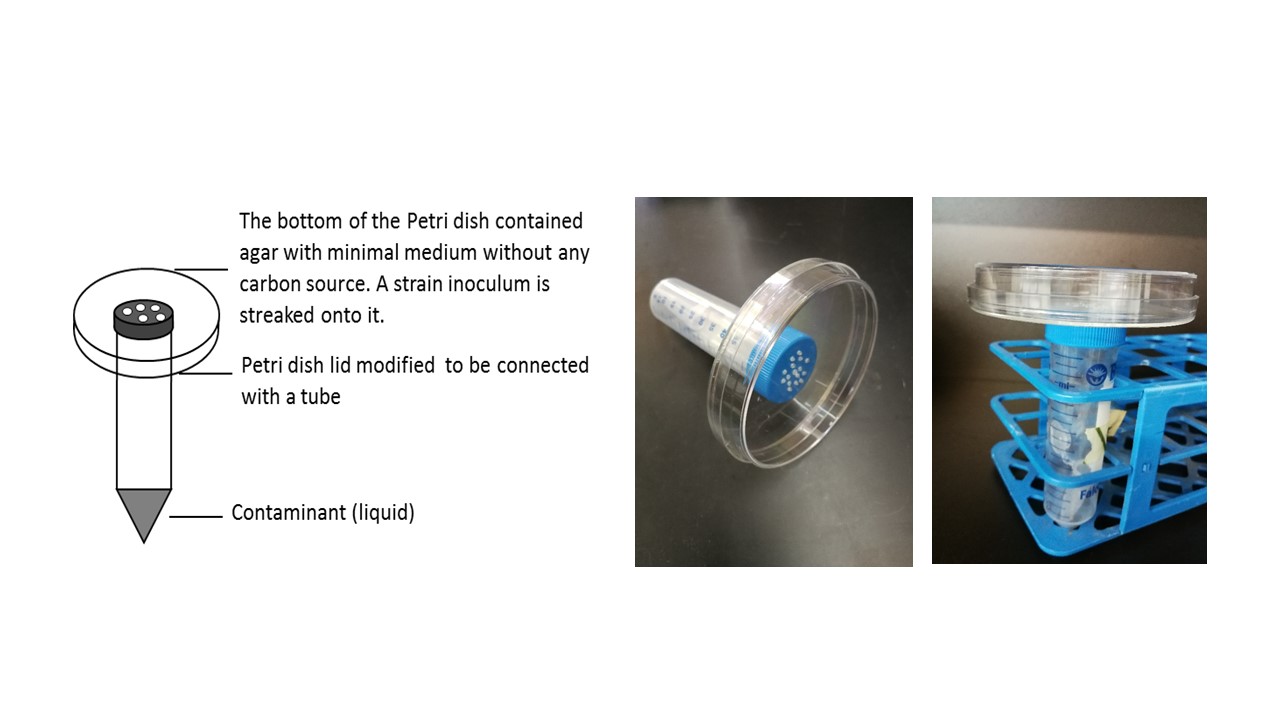

Supplement: Supplementary file 2 — Growth in gas phase via saturated atmosphere. (JPG 88 kb) [file 12864_2019_5677_MOESM2_ESM.jpg]
