# Supplementary material for: New insights into the genome of Rhodococcus ruber strain Chol-4
Source: BMC Genomics. 2019 May 2;20:332. doi: 10.1186/s12864-019-5677-2 (PMC6498646; doi:10.1186/s12864-019-5677-2)
Supplement: Supplementary file 3 — Table S2. Primers used in this work. (DOCX 13 kb) [file 12864_2019_5677_MOESM3_ESM.docx]

**Additional file 3: Table S2.** Primers used in this work

| Primer | Sequence | PCR conditions | Use |
| --- | --- | --- | --- |
| CH407  CH408 | AGATCTCCCGGGGAATTCGGTCGAGCTCGCCCGGTTCGAC  AGTACTGATATCTCTAGATCGTTCTCGAAGGGGGTGGCCT | Tm 56 °C, 1.5 min, 30 cycles. | Amplification of fragment A (up end *EcoR*I*-Xba*I*)* |
| CH409  CH410 | AGTACTGATATCTCTAGACCCAATACGGCGGAGCAGCAGGAA  GCTAGCCTGCAGAAGCTTATCCTGGAACGGCCGGGTGAACTC | Tm 60 °C, 1.5 min, 30 cycles. | Amplification of fragment B (down end *Xba*I-*Hind*III*)* |
| CH411  CH412 | AGATCTCCCGGGGAATTCGTCGACCGCGGCGATGTCCACC  AGTACTGATATCTCTAGACTCTACGTGCAACAGGGCGCAA | Tm 56 °C, 1.5 min, 30 cycles. | Amplification of fragment C (up end *Xho*I*-Hind*III) |
| CH413  CH414 | AGTACTGATATCTCTAGACTGCTCCGGCGTGAGCCGACG  GCTAGCCTGCAGAAGCTTCCGTCGTGCGCGAGGGGCTTTC | Tm 56 °C, 1.5 min, 30 cycles. | Amplification of fragment D ( down end *Xba*I-*Hind*III) |
| CH438  CH439 | TGTACTTCCCCGACGACCCGAT  TCCTCGCTCACCTCGCCGACTT | Tm 58 °C, 1.5 min, 30 cycles | Checking ketoadipate cluster deletion |
