# Supplementary material for: New insights into the genome of Rhodococcus ruber strain Chol-4
Source: BMC Genomics. 2019 May 2;20:332. doi: 10.1186/s12864-019-5677-2 (PMC6498646; doi:10.1186/s12864-019-5677-2)
Supplement: Supplementary file 4 — Figure S1. Sequence length vs GC content of the 129 scaffolds obtained in the initial assembly of R. ruber Chol-4 genome. (DOCX 428 kb) [file 12864_2019_5677_MOESM4_ESM.docx]

**Additional file 4: Figure S2.**

**
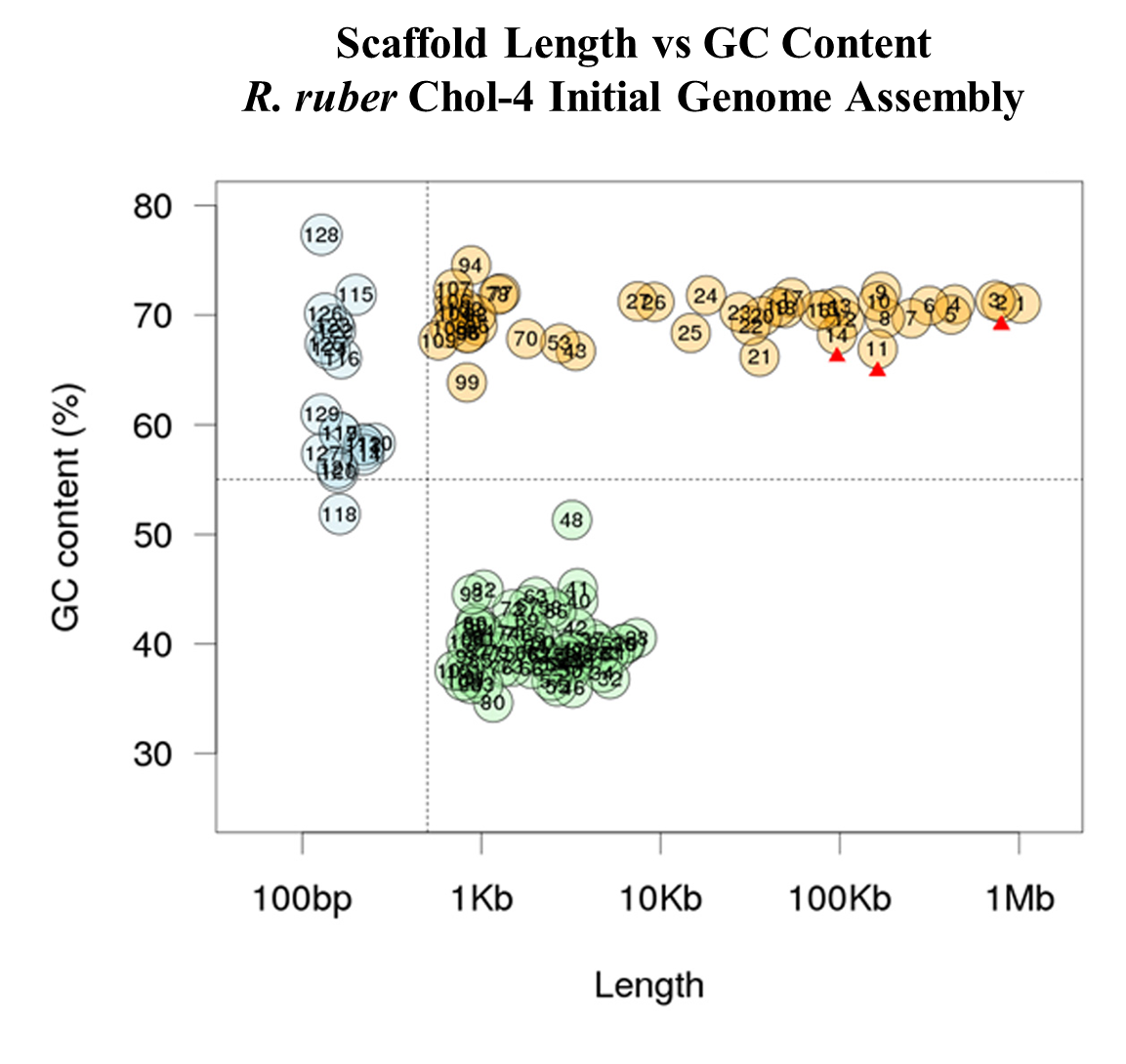
**

**Additional file 4: Figure S2. Sequence length vs GC content of the 129 scaffolds obtained in the initial assembly of *R. ruber* Chol-4 genome.** In the x-axis, the scaffold length (log scale). In the y-axis, the GC content (%). Every dot represents a DNA scaffold, numerated from the largest (Scaffold 1, 1025475bp) to shortest (Scaffold 129, 128bp). Most scaffolds contained a single continuous sequence contig, except number 2, 11 and 14 (indicated red triangles), which contained internal gaps. In blue, scaffolds shorter than 500bp (vertical dashed line) removed from the final assembly. In green, scaffolds with low GC content (below 55%, horizontal dashed line) that were removed due to their homology to other species genomes (mostly *A. thaliana*). Scaffold 48, also removed, had slightly higher GC content but it was practically identical to the transient expression vector pXGFP-P (GenBank FJ905213.1). In orange, the 44 scaffolds with high GC content and homology with the genome of other *Rhodococcus* species, that were included in the final assembly (GenBank NZ_ANGC00000000.2).
