# Supplementary material for: New insights into the genome of Rhodococcus ruber strain Chol-4
Source: BMC Genomics. 2019 May 2;20:332. doi: 10.1186/s12864-019-5677-2 (PMC6498646; doi:10.1186/s12864-019-5677-2)
Supplement: Supplementary file 5 — Quast genome assembly evaluation. (PDF 30 kb) [file 12864_2019_5677_MOESM5_ESM.pdf]

Report

	selected_contigs
# contigs (>= 0 bp)	44
# contigs (>= 1000 bp)	32
# contigs (>= 5000 bp)	27
# contigs (>= 10000 bp)	25
# contigs (>= 25000 bp)	23
# contigs (>= 50000 bp)	17
Total length (>= 0 bp)	5464707
Total length (>= 1000 bp)	5455287
Total length (>= 5000 bp)	5444882
Total length (>= 10000 bp)	5428193
Total length (>= 25000 bp)	5395546
Total length (>= 50000 bp)	5169916
# contigs	44
Largest contig	1025475
Total length	5464707
GC (%)	70.66
N50	438623
N75	178646
L50	4
L75	8
# N's per 100 kbp	18.65

All statistics are based on contigs of size >= 500 bp, unless otherwise noted (e.g., "# contigs (>= 0 bp)" and "Total length (>= 0 bp)" include all contigs).

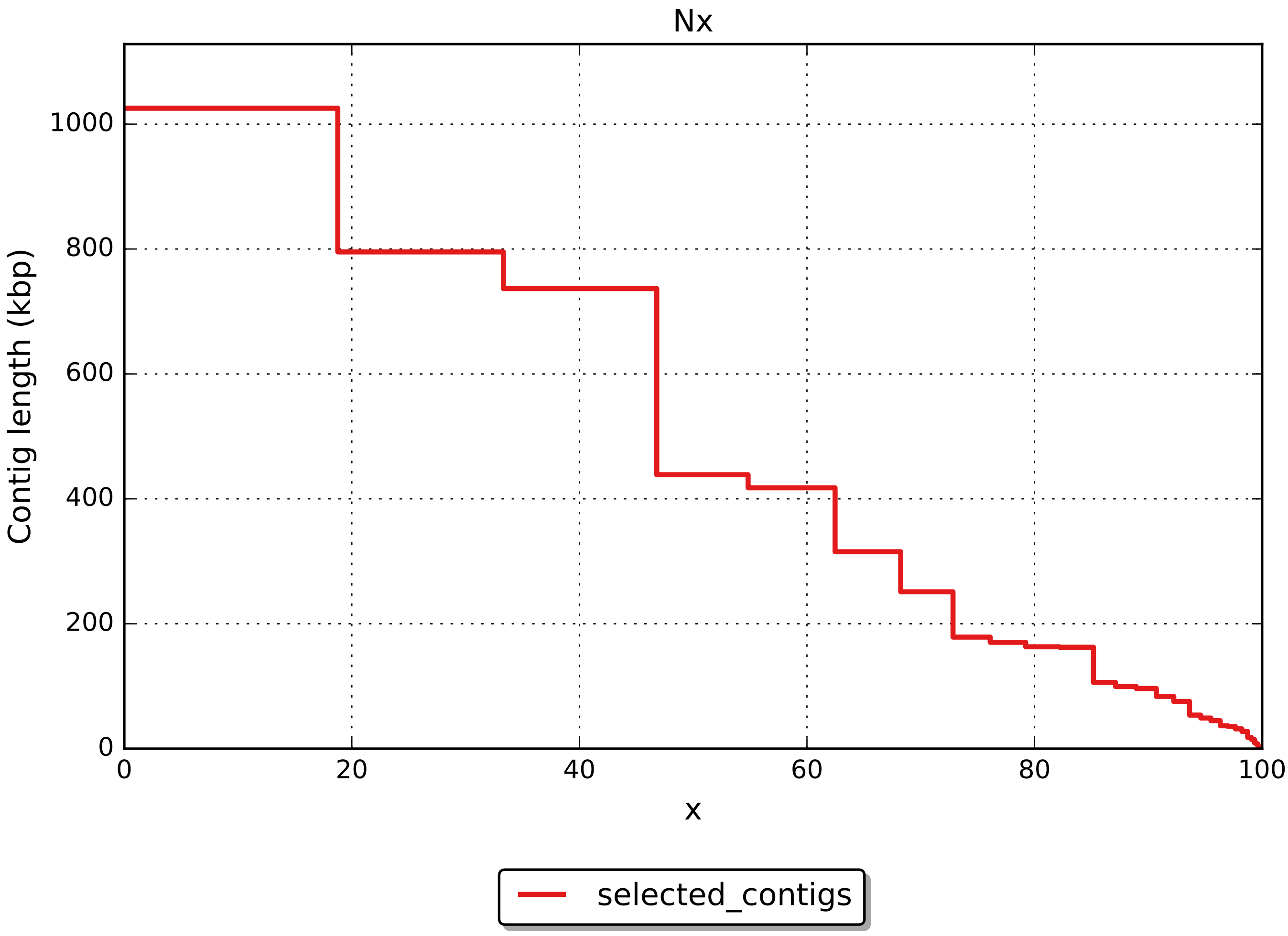

Nx
Contig length (kbp)
0
200
400
600
800
1000
0
20
40
60
80
100
x
selected_contigs

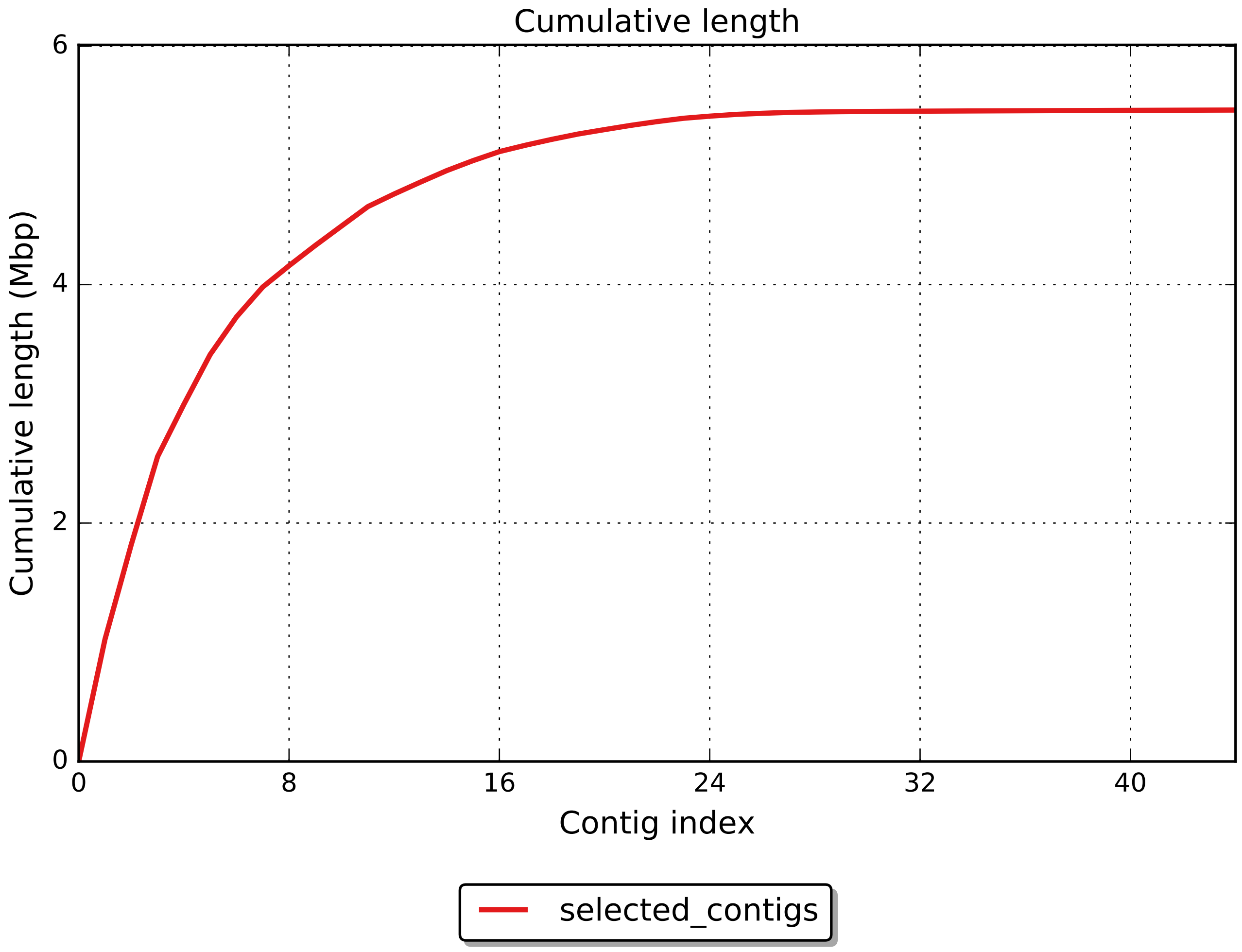

Cumulative length
Cumulative length (Mbp)
0
2
4
6
0
8
16
24
32
40
Contig index
selected_contigs

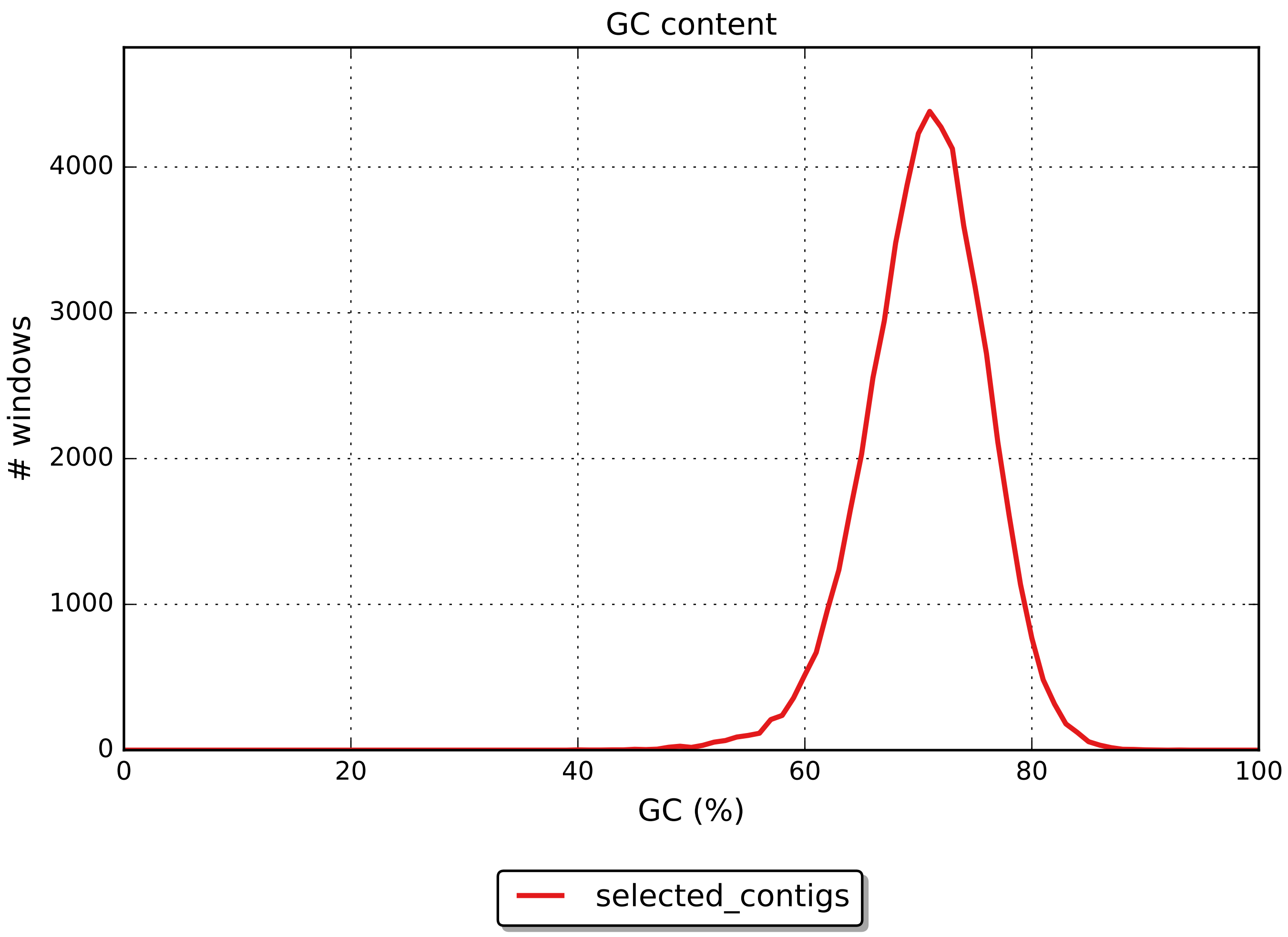
GC content
windows
0
1000
2000
3000
4000
0
20
40
60
80
100
GC (%)
selected_contigs

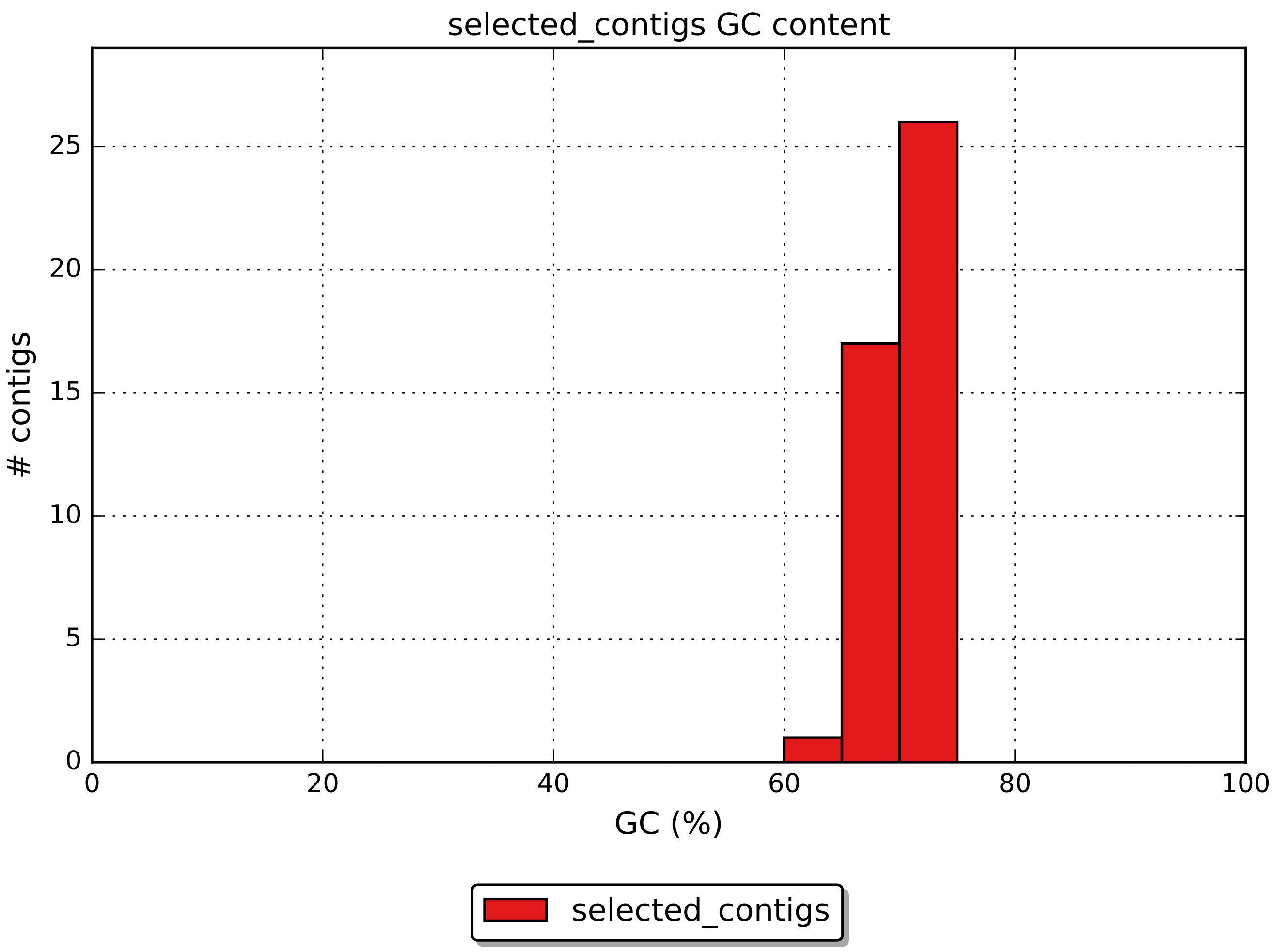
selected_contigs GC content
contigs
0
5
10
15
20
25
0
20
40
60
80
100
GC (%)
selected_contigs

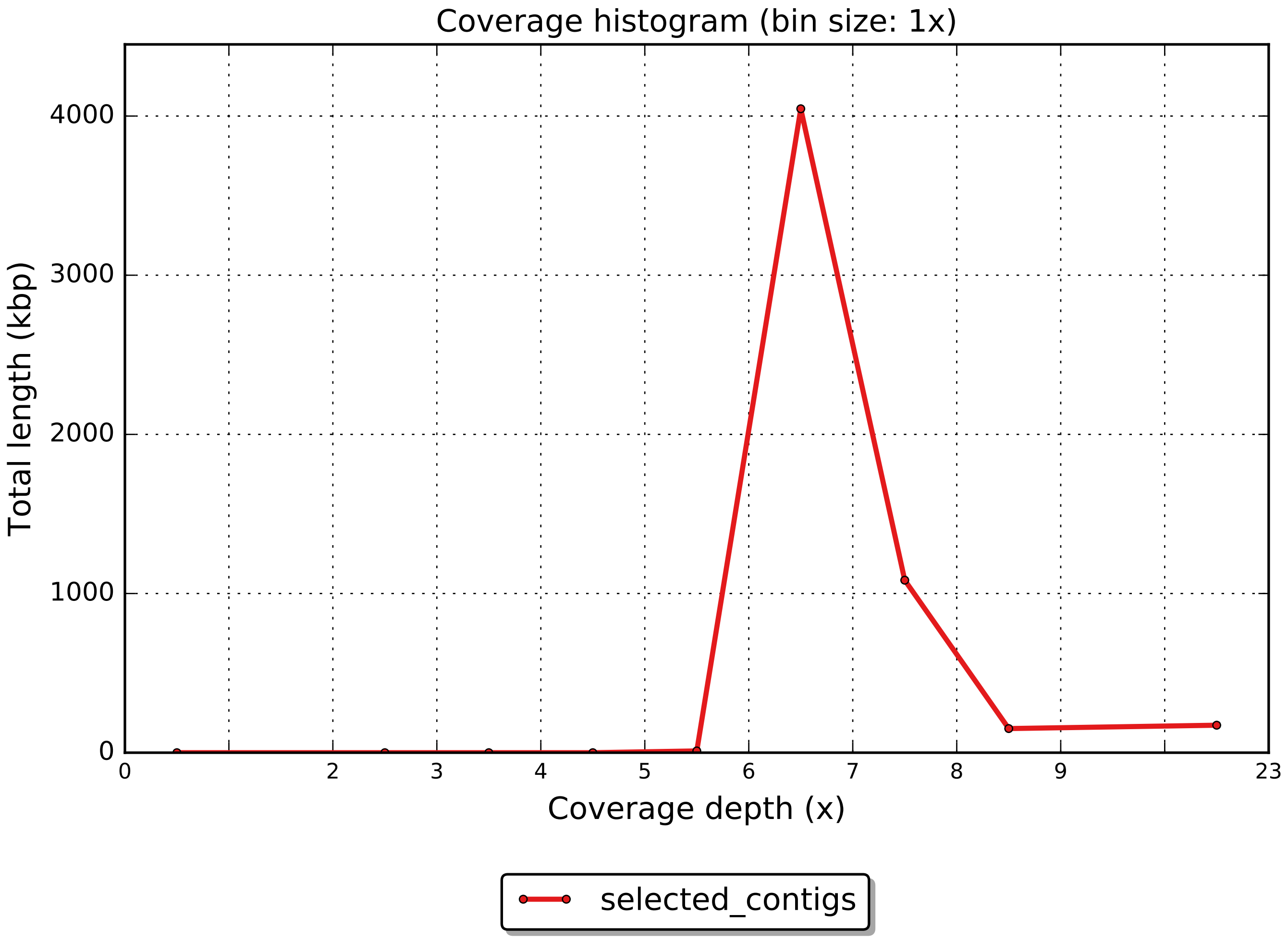

Coverage histogram (bin size: 1x)
Total length (kbp)
0
1000
2000
3000
4000
0
2
3
4
5
6
7
8
9
23
Coverage depth (x)
selected_contigs

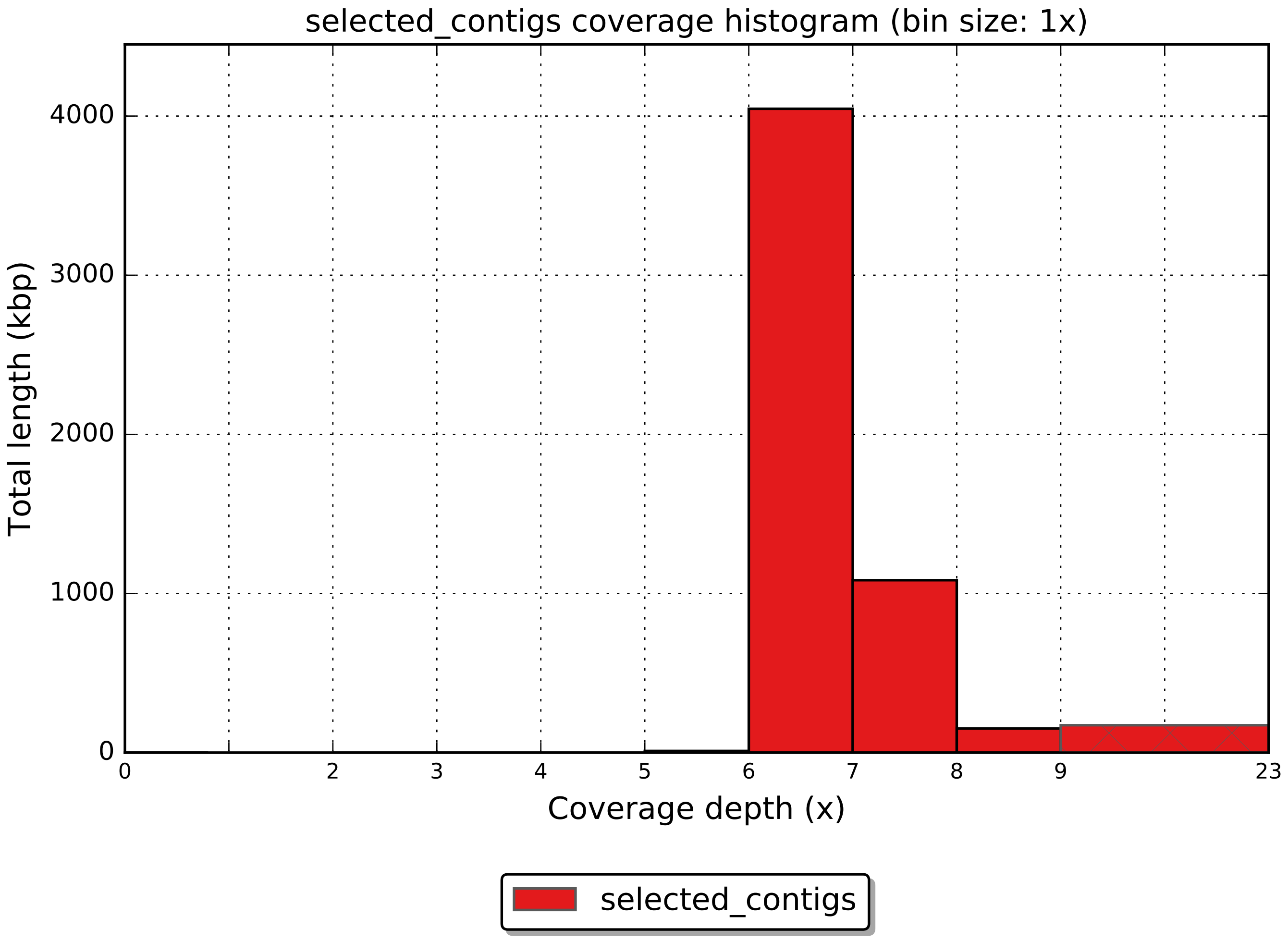

selected_contigs coverage histogram (bin size: 1x)
Total length (kbp)
0
1000
2000
3000
4000
0
2
3
4
5
6
7
8
9
23
Coverage depth (x)
selected_contigs
