# Supplementary material for: New insights into the genome of Rhodococcus ruber strain Chol-4
Source: BMC Genomics. 2019 May 2;20:332. doi: 10.1186/s12864-019-5677-2 (PMC6498646; doi:10.1186/s12864-019-5677-2)
Supplement: Supplementary file 6 — Table S3. Anticodons encoded in the R. ruber Chol-4 genome. (DOCX 20 kb) [file 12864_2019_5677_MOESM6_ESM.docx]

**Additional file 6: Table S3.** Anticodons encoded in the *R. ruber* Chol-4 genome.

| **Num. tRNAs** | **Amino acid** | **Cd.** | **Ant. Cd.** | **Contig** | **Start** | **End** | **L** | **Locus tag** |
| --- | --- | --- | --- | --- | --- | --- | --- | --- |
| 1 | Alanine (A) | GCC | tRNA-Ala-GGC | NZ_ANGC02000002.1 | 187388 | 187460 | 73 | D092_RS05650 |
| 2 | Alanine (A) | GCC | tRNA-Ala-GGC | NZ_ANGC02000002.1 | 187537 | 187609 | 73 | D092_RS05655 |
| 3 | Alanine (A) | GCG | tRNA-Ala-CGC | NZ_ANGC02000001.1 | 422777 | 422852 | 73 | D092_RS02040 |
| 4 | Alanine (A) | GCA | tRNA-Ala-TGC | NZ_ANGC02000005.1 | 411740 | 411815 | 73 | D092_RS15550 |
|  | Alanine (A) | GCT | - |  |  |  |  |  |
| 5 | Arginine (R) | CGG | tRNA-Arg-CCG | NZ_ANGC02000022.1 | 2562 | 2487 | 73 | D092_RS24260 |
| 6 | Arginine (R) | CGT | tRNA-Arg-ACG | NZ_ANGC02000023.1 | 22641 | 22713 | 73 | D092_RS24510 |
| 7 | Arginine (R) | AGA | tRNA-Arg-TCT | NZ_ANGC02000003.1 | 32114 | 32038 | 74 | D092_RS08525 |
| 8 | Arginine (R) | AGG | tRNA-Arg-CCT | NZ_ANGC02000001.1 | 338994 | 338922 | 73 | D092_RS01630 |
|  | Arginine (R) | CGC | - | - | - | - | - |  |
|  | Arginine (R) | CGA | - | - | - | - | - |  |
| 9 | Asparagine (N) | AAC | tRNA-Asn-GTT | NZ_ANGC02000002.1 | 339525 | 339597 | 73 | D092_RS06395 |
|  | Asparagine (N) | AAT | - | - | - | - | - |  |
| 10 | Aspartic acid (D) | GAC | tRNA-Asp-GTC | NZ_ANGC02000001.1 | 170705 | 170778 | 74 | D092_RS00775 |
| 11 | Aspartic acid (D) | GAC | tRNA-Asp-GTC | NZ_ANGC02000001.1 | 170964 | 171037 | 74 | D092_RS00785 |
|  | Aspartic acid (D) | GAT | - | - | - | - | - |  |
| 12 | Cysteine (C) | TGC | tRNA-Cys-GCA | NZ_ANGC02000003.1 | 400974 | 400904 | 71 | D092_RS10200 |
|  | Cysteine (C) | TGT | - | - | - | - | - |  |
| 13 | Glutamic acid (E) | GAG | tRNA-Glu-CTC | NZ_ANGC02000002.1 | 273884 | 273959 | 73 | D092_RS06065 |
| 14 | Glutamic acid (E) | GAG | tRNA-Glu-CTC | NZ_ANGC02000002.1 | 274019 | 274094 | 73 | D092_RS06070 |
| 15 | Glutamic acid (E) | GAA | tRNA-Glu-TTC | NZ_ANGC02000001.1 | 170575 | 170647 | 73 | D092_RS00770 |
| 16 | Glutamine (Q) | CAG | tRNA-Gln-CTG | NZ_ANGC02000002.1 | 273720 | 273791 | 72 | D092_RS06060 |
| 17 | Glutamine (Q) | CAA | tRNA-Gln-TTG | NZ_ANGC02000001.1 | 487465 | 487394 | 72 | D092_RS02330 |
| 18 | Glycine (G) | GGG | tRNA-Gly-CCC | NZ_ANGC02000004.1 | 21346 | 21416 | 71 | D092_RS1178 |
| 19 | Glycine (G) | GGA | tRNA-Gly-TCC | NZ_ANGC02000003.1 | 74274 | 74201 | 71 | D092_RS08710 |
| 20 | Glycine (G) | GGC | tRNA-Gly-GCC | NZ_ANGC02000003.1 | 400548 | 400473 | 73 | D092_RS10180 |
| 21 | Glycine (G) | GGC | tRNA-Gly-GCC | NZ_ANGC02000003.1 | 400766 | 400691 | 73 | D092_RS10190 |
| 22 | Glycine (G) | GGC | tRNA-Gly-GCC | NZ_ANGC02000003.1 | 401072 | 400997 | 73 | D092_RS10205 |
|  | Glycine (G) | GGT | - | - | - | - | - |  |
| 23 | Histidine (H) | CAC | tRNA-His-GTG | NZ_ANGC02000003.1 | 4850 | 4775 | 73 | D092_RS08395 |
|  | Histidine (H) | CAT | - | - | - | - | - |  |
| 24 | Isoleucine (I) | ATC | tRNA-Ile-GAT | NZ_ANGC02000005.1 | 411528 | 411601 | 74 | D092_RS15545 |
|  | Isoleucine (I) | ATT | - | - | - | - | - |  |
|  | Isoleucine (I) | ATA | - | - | - | - | - |  |
| 25 | Leucine (L) | CTG | tRNA-Leu-CAG | NZ_ANGC02000014.1 | 28859 | 28944 | 83 | D092_RS22275 |
| 26 | Leucine (L) | TTA | tRNA-Leu-TAA | NZ_ANGC02000001.1 | 530162 | 530238 | 74 | D092_RS02525 |
| **Num. tRNAs** | **Amino acid** | **Cd.** | **Ant. Cd.** | **Contig** | **Start** | **End** | **L** | **Locus tag** |
| 27 | Leucine (L) | CTA | tRNA-Leu-TAG | NZ_ANGC02000018.1 | 31076 | 30992 | 82 | D092_RS23655 |
| 28 | Leucine (L) | CTC | tRNA-Leu-GAG | NZ_ANGC02000003.1 | 625844 | 625759 | 86 | D092_RS11260 |
| 29 | Leucine (L) | CTC | tRNA-Leu-GAG | NZ_ANGC02000003.1 | 626122 | 626037 | 86 | D092_RS11265 |
| 30 | Leucine (L) | TTG | tRNA-Leu-CAA | NZ_ANGC02000002.1 | 611221 | 611145 | 74 | D092_RS07605 |
|  | Leucine (L) | CTT | - | - | - | - | - |  |
| 31 | Lysine (K) | AAA | tRNA-Lys-TTT | NZ_ANGC02000001.1 | 169593 | 169521 | 73 | D092_RS00760 |
| 32 | Lysine (K) | AAG | tRNA-Lys-CTT | NZ_ANGC02000003.1 | 1414 | 1342 | 73 | D092_RS08380 |
| 33 | Methionine (M) | ATG | tRNA-Met-CAT | NZ_ANGC02000002.1 | 170324 | 170400 | 74 | D092_RS05550 |
| 34 | Methionine (M) | ATG | tRNA-Met-CAT | NZ_ANGC02000002.1 | 339811 | 339887 | 74 | D092_RS06400 |
| 35 | Methionine (M) | ATG | tRNA-Met-CAT | NZ_ANGC02000006.1 | 139552 | 139477 | 73 | D092_RS16200 |
| 36 | Methionine (M) | ATG | tRNA-Met-CAT | NZ_ANGC02000008.1 | 69116 | 69040 | 74 | D092_RS18695 |
| 37 | Phenylalanine (F) | TTC | tRNA-Phe-GAA | NZ_ANGC02000001.1 | 170816 | 170892 | 74 | D092_RS00780 |
|  | Phenylalanine (F) | TTT | - | - | - | - | - |  |
| 38 | Proline (P) | CCG | tRNA-Pro-CGG | NZ_ANGC02000007.1 | 36608 | 36681 | 74 | D092_RS17315 |
| 39 | Proline (P) | CCA | tRNA-Pro-TGG | NZ_ANGC02000003.1 | 74412 | 74488 | 74 | D092_RS08715 |
| 40 | Proline (P) | CCC | tRNA-Pro-GGG | NZ_ANGC02000002.1 | 737857 | 737930 | 74 | D092_RS08120 |
|  | Proline (P) | CCT | - | - | - | - | - |  |
| 41 | Serine (S) | AGC | tRNA-Ser-GCT | NZ_ANGC02000023.1 | 22478 | 22569 | 89 | D092_RS24505 |
| 42 | Serine (S) | TCA | tRNA-Ser-TGA | NZ_ANGC02000023.1 | 16180 | 16267 | 85 | D092_RS24475 |
| 43 | Serine (S) | TCG | tRNA-Ser-CGA | NZ_ANGC02000019.1 | 6341 | 6428 | 88 | D092_RS23745 |
| 44 | Serine (S) | TCC | tRNA-Ser-GGA | NZ_ANGC02000007.1 | 248085 | 248172 | 85 | D092_RS18320 |
|  | Serine (S) | AGT | - | - | - | - | - |  |
|  | Serine (S) | TCT | - | - | - | - | - |  |
| 45 | Threonine (T) | ACA | tRNA-Thr-TGT | NZ_ANGC02000001.1 | 5702 | 5776 | 72 | D092_RS00030 |
| 46 | Threonine (T) | ACC | tRNA-Thr-GGT | NZ_ANGC02000006.1 | 139669 | 139597 | 73 | D092_RS16205 |
| 47 | Threonine (T) | ACG | tRNA-Thr-CGT | NZ_ANGC02000007.1 | 1059 | 984 | 73 | D092_RS17135 |
|  | Threonine (T) | ACT | - | - | - | - | - |  |
| 48 | Tryptophan (W) | TGG | tRNA-Trp-CCA | NZ_ANGC02000006.1 | 138001 | 137926 | 73 | D092_RS16180 |
| 49 | Tyrosine (Y) | TAC | tRNA-Tyr-GTA | NZ_ANGC02000006.1 | 139981 | 139896 | 83 | D092_RS16210 |
|  | Tyrosine (Y) | TAT | - | - | - | - | - |  |
| 50 | Valine (V) | GTA | tRNA-Val-TAC | NZ_ANGC02000002.1 | 358060 | 358134 | 72 | D092_RS06490 |
| 51 | Valine (V) | GTC | tRNA-Val-GAC | NZ_ANGC02000003.1 | 400683 | 400609 | 72 | D092_RS10185 |
| 52 | Valine (V) | GTC | tRNA-Val-GAC | NZ_ANGC02000003.1 | 400902 | 400828 | 72 | D092_RS10195 |
| 53 | Valine (V) | GTG | tRNA-Val-CAC | NZ_ANGC02000003.1 | 401383 | 401454 | 72 | D092_RS10210 |
|  | Valine (V) | GTT | - | - | - | - | - |  |
|  | Stop codon | TGA | - |  |  |  |  |  |
|  | Stop codon | TAG | - |  |  |  |  |  |
|  | Stop codon | TAA | - |  |  |  |  |  |

**Cd**.: codon; **Ant. Cd.**: anticodon; **L**: length
