# Supplementary material for: New insights into the genome of Rhodococcus ruber strain Chol-4
Source: BMC Genomics. 2019 May 2;20:332. doi: 10.1186/s12864-019-5677-2 (PMC6498646; doi:10.1186/s12864-019-5677-2)
Supplement: Supplementary file 7 — Table S4. List of mobile elements found in the R. ruber Chol-4 genome. (DOCX 25 kb) [file 12864_2019_5677_MOESM7_ESM.docx]

**Additional file 7: Table S4.** List of mobile elements found in the *R. ruber* Chol-4 genome.

| **Contig** | **Locus** | **Location**  Complement | **Function** | **Max identity; Size; % identity/ % Similarity** |
| --- | --- | --- | --- | --- |
| NZ_ANGC02000001.1 | D092_RS04160 | 894293..89596 | Plasmid pria4b orf-3 family protein (phage integrase) | Plasmid pria4b ORF-3 family protein [*Rhodococcus rhodochrous* ATCC 21198] ETT25674.1;593aa |
|  |  |  |  | 479/555(86%); 509/555(91%) |
|  | D092_RS04185 | 905171..905756 | Transposase (pseudogen) |  |
|  | D092_RS04190 | 905769..906277 | Transposase (pseudogen |  |
|  | D092_RS04720 | 1021617..1022753 | Phage integrase , site-specific tyrosine recombinase pham107 | Integrase [*Gordonia aichiensis*] WP_005179717; 369aa |
|  |  |  |  | 237/367(65%); 278/367(75%) |
|  | D092_RS04730 | 1023799..1024926 | Hypothetical protein;DNA helicase, phage-associated #pham380; repa | Hypothetical protein [*Nocardia sp*. NRRL WC-3656] WP_030515513.1; 333aa |
|  |  |  |  | 196/322(61%); 241/322(74%) |
| NZ_ANGC02000002.1 | D092_RS07855 | 667672..668442 | ATP-binding protein/transposition helper | ATP-binding protein [*Mycobacterium kumamotonense*] WP_065289691.1; 255aa |
|  |  |  |  | 232/253(92%); 244/253(96%) |
|  | D092_RS07860 | 668439..669950 | Mobile element protein | Transposase [*Mycobacterium sp.* YC-RL4] ANE81404.1; 505aa |
|  |  |  |  | 409/501(82%); 442/501(88%) |
|  | D092_RS08235 | 765763..766713 | Putative integrase/recombinase | Recombinase [*Gordonia sp.* SGD-V-85] KSU51113.1; 345aa |
|  |  |  |  | 315/316(99%); 316/316(100%) |
|  | D092_RS08280 | 773291..776302 | Dde transposase | DDE transposase [*Gordonia terrae*] WP_004018622.1; 1003aa |
|  |  |  |  | 1002/1003(99%); 1003/1003(100%) |
| NZ_ANGC02000003.1 | D092_RS09470 | 240258..241452 | Transposase (pseudogen) | Transposase [*Pseudonocardia asaccharolytica*] WP_028932083.1; 138aa  127/138(92%); 132/138(95%)  Transposase [*Nocardia elegans*] WP_063023642.1; 350aa  328/350(94%); 338/350(96%)  Integrase [*Mycobacterium fortuitum*] WP_061264304.1; 377aa  174/348(50%); 213/348(61%) |
|  | D092_RS09475 | 241478..241948 | Mobile element protein: transposase |  |
|  | D092_RS10710 | 506067..507119  652545..653603 | 3-keto-5-aminohexanoate cleavage protein//transposase  Integrase |  |
|  |  |  |  |  |
|  | D092_RS11385 |  |  |  |
| NZ_ANGC02000004.1 | D092_RS11825 | 31773..32825 | 3-keto-5-aminohexanoate cleavage protein//transposase | Transposase [*Nocardia salmonicida*] WP_062985089.1; 350aa |
|  |  |  |  | 317/350(91%); 334/350(95%) |
|  | D092_RS13055 | 299545..301230 | Mobile element protein | Transposase [*Mycobacterium tusciae*] WP_040538977.1; 577aa |
|  |  |  |  | 316/597(53%); 371/597(62%) |
| NZ_ANGC02000005.1 | D092_RS15555 | 411922..412770 | Site-sepcific integrase | Site-specific integrase [*Nocardia testacea*] WP_051165467.1; 410aa |
|  |  |  |  | 165/312(53%); 207/312(66%) |
| NZ_ANGC02000007.1 | D092_RS17655 | 107090..108331 | Mobile element protein; integrase | Integrase [*Mycobacterium intracellulare*] WP_009954429.1; 417aa |
|  |  |  |  | 288/413(70%); 324/413(78%) |
|  | D092_RS17945 | 168554..169801 | Mobile element protein: transposase: is*1164* | Transposase [*Mycobacterium sp.* NAZ190054] KWX61743.1; 412aa |
|  |  |  |  | 339/404(84%); 367/404(90%) |
|  | D092_RS17950 | 169852..170079 | Dna resolvase/recombinase(pseudogen) |  |
|  |  |  |  |  |
|  | D092_RS17975 | 175058..177511 | Hypothethical protein; putative DNA integrase/recombinase | Integrase [*Mycobacterium*] WP_046182709.1; 821aa484/792(61%); 574/792(72%) |
|  | D092_RS17980 | 177508..178743 | Putative DNA integrase/recombinase | Integrase [*Mycobacterium kumamotonense*] OBY29445.1; 395aa |
|  |  |  |  | 277/390(71%); 313/390(80%) |
|  | D092_RS18300 | 243517..244764 | Mobile element protein: transposase: is*1164* | Transposase [*Mycobacterium sp.* NAZ190054] KWX61743.1; 412aa |
|  |  |  |  | 339/404(84%); 367/404(90%) |
| NZ_ANGC02000011.1 | D092_RS20485 | 7786..9111 | Mobile element protein: transposase | Transposase [*Propionibacterium freudenreichii*] WP_048769214.1; 441aa |
|  |  |  |  | 440/441(99%); 440/441(99%) |
|  | D092_RS20525 | 16392..17669 | Mobile element protein: transposase | Transposase [Jiangella muralis] WP_053208317.1; 416aa |
|  |  |  |  | 387/421(92%); 399/421(94%) |
|  | D092_RS20545 | 19950..20843 | Integrase | Integrase [*Streptomyces zinciresistens*] WP_007505190; 296 aa |
|  |  |  |  | 237/297(80%); 255/297(85%) |
|  | D092_RS20550 | 20840..21169 | Mobile element protein: transposase | Transposase [*Streptomyces sp.* NBRC 110035]; WP_042172217.1; 109 aa |
|  |  |  |  | 82/109(75%); 91/109(83%) |
|  | D092_RS20565 | 22392..23345 | Putative integrase/recombinase | Recombinase [*Nocardia farcinica*] WP_060594881.1; 317aa |
|  |  |  |  | 316/317(99%); 316/317(99%) |
|  | D092_RS20610 | 29738..32749 | Dde transposase | DDE transposase [*Gordonia terrae*] WP_004018622.1; 1003aa |
|  |  |  |  | 974/1003(97%); 987/1003(98%) |
|  | D092_RS20840 | 90718..91287 | Phage protein | Hypothetical protein HMPREF0305_12394 [Corynebacterium pseudogenitalium ATCC 33035] EFQ79380.1; 137 aa |
|  |  |  |  | 69/137(50%); 87/137(63%) |
|  | D092_RS20850 | 93198..93815 | Resolvase/integrase bin | Putative resolvase [*Nocardia farcinica* IFM 10152] BAD60721.1; 222aa |
|  |  |  |  | 197/205(96%); 200/205(97%) |
|  | D092_RS20875 | 97358..100372 | Dde transposase | DDE transposase [*Nocardia farcinica*] WP_011212398.1; 1005aa |
|  |  |  |  | 971/1005(97%); 984/1005(97%) |
|  | D092_RS20895 | 103583..105511 | Phage integrase | Phage integrase (modular protein) [*Tetrasphaera australiensis* Ben110] CCH73768.1; 842aa. |
|  |  |  |  | 326/641(51%); 427/641(66%) |
|  | D092_RS21010 | 127468..127950 | Transposase, pseudogen | Putative integrase [*Gordonia sihwensis* NBRC 108236] GAC62749.1; 398aa |
|  |  |  |  | 257/390(66%); 302/390(77%) |
| NZ_ANGC02000014.1 | D092_RS22495 | 78212..79378 | Integrase | Transposase [*Nocardia elegans*] WP_063031757.1;  333aa282/332(85%); 301/332(90%) |
|  | D092_RS22515 | 83731..84732 | Mobile element protein: transposase | Transposase [*Corynebacterium maris*] WP_020935232.1; 384aa |
|  |  |  |  | 172/262(66%); 195/262(74%) |
| NZ_ANGC02000020.1 | D092_RS24085 | 35960..>36759 | Mobile element protein: transposase | Transposase [*Corynebacterium maris]* WP_020935232.1; 384aa |
|  |  |  |  | 172/262(66%); 195/262(74%) |
| NZ_ANGC02000021.1 | D092_RS24110 | 5698..7239 | Hypothetical protein; mobile element protein | Integrase [*Mycobacterium heraklionense*] WP_047318348.1; 513aa |
|  |  |  |  | 379/513(74%); 411/513(80%) |
|  | D092_RS24115 | 7306..8070 | Atp-binding protein; mobile element protein | Transposase [*Mycobacterium canettii*] WP_015294405.1; 251aa |
|  |  |  |  | 213/252(85%); 233/252(92%) |
|  | D092_RS24135 | 12110..13276 | Integrase | Putative integrase [*Gordonia sihwensis* NBRC 108236] GAC62749.1; 398aa |
|  |  |  |  | 259/386(67%); 300/386(77%) |
| NZ_ANGC02000028.1 | D092_RS24680 | 342..1487 | Putative integrase/recombinase | Integrase [*Arthrobacter sp*. H14] WP_026536562.1; 365aa |
|  |  |  |  | 229/361(63%); 274/361(75%) |

Mobile elements were identified using the RAST server. Identities were searched using the NCBI Blast server (<https://www.ebi.ac.uk/Tools/sss/ncbiblast/> or https://blast.ncbi.nlm.nih.gov/Blast.cgi).
