# Supplementary material for: New insights into the genome of Rhodococcus ruber strain Chol-4
Source: BMC Genomics. 2019 May 2;20:332. doi: 10.1186/s12864-019-5677-2 (PMC6498646; doi:10.1186/s12864-019-5677-2)
Supplement: Supplementary file 8 — Table S5. List of recombinases identified in the R. ruber Chol-4 genome. (DOCX 19 kb) [file 12864_2019_5677_MOESM8_ESM.docx]

**Additional file 8: Table S5.** List of recombinases identified in the *R. ruber* Chol-4 genome.

| **Conting** | **Locus Tag** | | **Localization** | **Protein Reference /size** | **Function** | **Max identity; Size; % identity; % Similarity** |
| --- | --- | --- | --- | --- | --- | --- |
| NZ_ANGC02000001.1 | D092_RS02770  D092_RS02875  D092_RS02865 | 581107..582270  607260..609128  600680..603973 | | KXF88421.1  387AA  KXF88019.1  622AA  KXF88017 1097AA | Rmuc family DNA recombination protein. The function of the rmuc protein is unknown but it is suspected that it is either a structural protein that protects  DNA against nuclease action, or is itself involved in DNA cleavage at the regions of DNA secondary structures  Exodeoxyribonuclease v subunit alpha;recombinase recd; exodeoxyribonuclease v subunit gamma: Double-strand break repair via homologous recombination ; recbcd-dependent single-strand annealing (SSA). | DNA recombinase [*Nocardia terpenica*] KZM71360.1; 378aa  266/382(70%); 300/382(78%)  Multispecies: exodeoxyribonuclease v subunit alpha [*Rhodococcus*] WP_054248544.1: 622aa  477/619(77%), 529/619(85%). |
|  |  |  |  |  |  |  |
| NZ_ANGC02000002.1 | D092_RS08360 | | 793035..794066 | KXF87439.1  643AA | DNA recombinase | Multispecies: DNA recombinase [*Rhodococcus*] WP_017682306.1; 343aa |
|  |  |  |  |  |  | 343/343(100%); 343/343(100%) |
|  | D092_RS08085 | | 731086..732003 | KXF87397.1  305AA | Recombinase xerd; site-specific tyrosine recombinase xercd:it functions in circular chromosome separation. | Site-specific tyrosine recombinase xerd [*Mycobacterium* sp. Ga-2829]; WP_059089360.1: 317aa  232/305(76%); 258/305(84%) |
|  | D092_RS07920 | | 693426..693779 | KXF87369  117AA | Hp;reca/rada recombinase: homologous recombination and underpin genome stability, by promoting the repair of double-stranded DNA breaks and the rescue of collapsed DNA replication fork. | Hypothetical protein [*Nocardia paucivorans*] WP_040790284.1; 113aa  98/117(84%); 105/117(89%) |
| NZ_ANGC02000003.1 | D092_RS11460 | | 670961..671842 | KXF86722.1  293AA | Recombinase recb;exonuclease recb: homologous recombination. | Recombinase b [*Mycobacterium abscessus*] cpv10095.1; 293aa |
|  |  |  |  |  |  | 178/282(63%) 210/282(74%) |
|  | D092_RS09725 | | 298516..299094 | KXF86416  192AA | Recombination regulator recx; recombinase recx: recx inhibits reca recombinase and coprotease. | Recombination regulator recx [*Nocardia otitidiscaviarum*] WP_029928300.1; 166aa111/168(66%); 129/168(76%) |
|  | D092_RS09720 | | 296375..298507 | KXF86415.1  710 AA | Recombinase reca, intein-containing: repair and maintenance of DNA | Recombinase reca, intein-containing [*Nocardia sp.* Bmg51109] WP_036567761.1; 711aa |
|  |  |  |  |  |  | 628/711(88%); 667/711(93%) |
|  | D092_RS09250 | | 192362..193288 | KXF86336.1  308AA | Recombinase xerc: site-specific tyrosine recombinase xercd. | Recombinase xerc [*Mycobacterium* sp. 852013-51886_sch5428379] obb61433.1; 300aa |
|  |  |  |  |  |  | 205/296(69%); 237/296(80%) |
| NZ_ANGC02000005.1 | D092_RS15475 | | 392877..394130 | KXF85732.1  417AA | Dna replication/repair protein recf;recombinase recf; (recfr, involved in recombination processes in mycobacteria) | Dna replication/repair protein recf *[Nocardia pseudovaccinii]* WP_063042415.1; 395aa  268/393(68%); 312/393(79%) |
| NZ_ANGC02000007.1 | D092_RS18120 | | 206614..207222 | KXF85034.1  202AA | Recombination protein recr;recombinase recr (recfr, involved in recombination processes in mycobacteria) | Recombination protein recr [*Nocardia* sp. Bmg111209] WP_019931781.1; 202aa  190/202(94%); 197/202(97%) |
|  | D092_RS17765 | | 131651..132259 | KXF84976.1  202AA | Recombination protein recr;recombinase recr | [repetido] |
| NZ_ANGC02000013.1 | D092_RS21935 | | 61919..63511 | KXF84177  530AA | Recombinase recb  Recbcd-dependent single-strand annealing (ssa) | Recombinase recb [*Nocardia tenerifensis*] WP_051186107.1; 537aa  334/529(63%); 394/529(74%) |
