# Supplementary material for: New insights into the genome of Rhodococcus ruber strain Chol-4
Source: BMC Genomics. 2019 May 2;20:332. doi: 10.1186/s12864-019-5677-2 (PMC6498646; doi:10.1186/s12864-019-5677-2)
Supplement: Supplementary file 9 — Table S6. List of restriction modification systems identified in the R. ruber Chol-4 genome. (DOCX 18 kb) [file 12864_2019_5677_MOESM9_ESM.docx]

**Additional file 9: Table S6.** List of restriction modification systems identified in the *R. ruber* Chol-4 genome.

| **Contig** | **Locus Tag** | **Localization** | **Protein reference and size** | **Function** | **Max ID source/aa id/pos id** |
| --- | --- | --- | --- | --- | --- |
| NZ_ANGC02000001.1 | D092_RS03120 | 656070..657014 | KXF88067  314aa | Mrr restriction system protein (endonuclease IV) | Restriction endonuclease [*Mycobacterium* sp. Ga-1199] WP_064350384.1; 295aa  149/296(50%); 197/296(66%) |
|  | D092_RS03645 | 771133..771903 | KXF88439  256aa | Endonuclease IV | Deoxyribonuclease IV [*Smaragdicoccus niigatensis*] WP_018159685.1; 257aa  183/253(72%); 212/253(83%) |
|  | D092_RS04610 | 994228..994896 | KXF88458  222aa | Deoxyribonuclease | Hypothetical protein duf1524 [*Nocardia nova* sh22a] ahh16003.1; 218aa  134/207(65%); 158/207(76%) |
| NZ_ANGC02000003.1 | D092_RS08630 | 58570..59193 | KXF86763  312aa | HNH endonuclease | Multispecies: HNH endonuclease [*Mycobacterium*] WP_023986061.1; 214aa  135/199(68%); 160/199(80%) |
|  | D092_RS11355 | 641913..646058 | KXF86705  1381aa | Endonuclease | Endonuclease [*Nocardia pseudovaccinii*] WP_063037236.1; 1403aa  855/1385(62%); 1015/1385(73%) |
|  | D092_RS09220 | 186939..187295 | KXF86330  118aa | Endonuclease | Hypothetical protein [*Nocardia grenadensis*] WP_063041706.1; 118aa  71/118(60%); 85/118(72%) |
| NZ_ANGC02000007.1 | D092_RS17260 | 26161..26955 | KXF84887  264aa | Endonuclease III | Endonuclease III [*Gordonia amarae*] WP_040514706.1; 268aa  209/257(81%); 222/257(86%) |
|  | D092_RS17490 | 71233..71598 | KXF84930  121aa | HNH endonuclease | Hnh endonuclease [*Modestobacter caceresii*] WP_036340911.1; 130aa  73/118(62%); 81/118(68%) |
|  | D092_RS18325 | 248313..249338 | KXF85067  341aa | Deoxyribonuclease | Deoxyribonuclease [*Gordonia* sp. Ucd-tk1] WP_065630560.1; 360aa  211/353(60%); 231/353(65%) |
| NZ_ANGC02000008.1 | D092_RS18900 | 107380..108993 | KXF84792  537aa | Type I restriction-modification system, DNA-methyltransferase subunit M (EC 2.1.1.72) | Type I restriction-modification system, DNA-methyltransferase subunit m [A*rthrobacter* sp. Pamc 25486] aiy01962.1; 539aa  424/538(79%); 466/538(86%) |
| NZ_ANGC02000010.1 | D092_RS19995 | 23280..24380 | KXF84462  366aa | HNH endonuclease | Hnh endonuclease [*Mycobacterium smegmatis*] WP_058125483.1; 359aa  211/352(60%); 254/352(72%) |
| NZ_ANGC02000011.1 | D092_RS20810 | 78083..81217 | KXF84373  1044aa | Type I restriction-modification system, restriction subunit R (EC 3.1.21.3) | Restriction endonuclease subunit R [*Rocardia arizonensis*] WP_054811818.1; 1044aa  965/1044(92%); 1009/1044(96%) |
|  | D092_RS20820 | 82443..84188 | KXF84375  581aa | restriction endonuclease subunit M (type I) | Restriction endonuclease subunit M [*Kutzneria* sp. 744] WP_043722299.1; 581aa  546/581(94%); 561/581(96%) |
| NZ_ANGC02000014.1 | D092_RS22520 | 85260..87227 | KXF84108  655aa | restriction endonuclease subunit S (Type I) | Restriction endonuclease subunit s [A*rthrobacter* sp. Yc-rl1] WP_047119061.1; 657aa505/647(78%); 570/647(88%) |
| NZ_ANGC02000018.1 | D092_RS23505 | 1752..2426 | KXF83805  224aa | Endonuclease | Endonuclease [*Gordonia araii*] WP_040521252.1; 227aa  198/227(87%); 208/227(91%) |
|  |  |  |  |  |  |
